# Supplementary material for: Review of psychological effects of dog bites in children
Source: BMJ Paediatr Open. 2024 Jun 6;8(1):e000922. doi: 10.1136/bmjpo-2020-000922 (PMC11163822; doi:10.1136/bmjpo-2020-000922)
Supplement: Supplementary data [file bmjpo-2020-000922supp001.pdf]

Supplementary Material: A review of psychological effects of dog bites in children

Westgarth, Carri., Provozza, Serena., Nicholas, Jade., Gray, Victoria.

Supplementary Table 1: Case studies/small case-series identified in searches of psychological consequences of dog bites to children.

**Paper title**	**Year**	**Author/s**	**Study design**	**Participants**	**Psychological findings**
Behavioural Assessment and Treatment of PTSD in Prepubertal Children: Attention to Developmental Factors and Innovative Strategies in the Case Study of a Family	1997	AM Albano<br>PP Miller<br>G Coté<br>DH Barlow	Mixed methods case study observing one family after a traumatic incident dog bite.<br>Prospective.<br>Diagnostic interviews.<br>Observational methods including rating sleep cycles of children, recording enuresis episodes and recording incidents of new behaviour since the incident.<br>Family behavioural tests with units of distress calculated.	N=4<br>M age 7<br>F age 6 (bitten)<br>F age 4<br>F age 2	Oldest 2 children diagnosed with PTSD.<br>Nocturnal changes in 2 children (bed wetting) represents a "significant change in functioning".<br>All 4 children experienced moderate to severe sleep disturbances.<br>"It was noted that as the age of the child increased, the more cognitively mediated symptoms and expression of fear became apparent."<br>Adult symptoms went into remission without treatment.
Selective mutism due to a dog bite trauma in a 4-year-old girl: a case report	2009	D Anyfantakis<br>E Botzakis<br>E Mplevrakis<br>EK Symvoulakis<br>I Arbiros	Case study.<br>Retrospective.	N=1<br>F age 5	On admission was confused and lethargic – findings showed hypovolemic shock.<br>2nd day of hospitalisation – child was withdrawn and depressed – used non verbal communication.<br>2 months after discharge was non-verbal and had reccurent traumatic memories.<br>Case met all criteria for selective mutism and acute PTSD.<br>Treatment consisted of "supportive psychotherapy for the child and consecutive sessions of counselling for her parents".<br>Improvement after 6M.

Clinical Round Table: A Case of Trauma to a 21-Month-Old Girl	1995	MJ Drell<br>TJ Gaensbauer<br>CH Siegel<br>M Sugar	Case study.<br>Retrospective.	N=1<br>F age 1	Detailed account of symptoms and therapy of child and mother.<br>Child displayed fearfulness of dogs, nightmares, anger, difficulty playing.
Comminuted mandibular fracture in child victim of dog bite	2012	M Furtado de Carvalho<br>LAP Hardtke<br>MF Cota de Souza<br>V de Oliveira Araujo	Case study.<br>Retrospective.	N=1<br>M age 3	The patient was seen every day by oral and maxillofacial surgeons, dieticians, pediatricians, plastic surgeons, ophthalmologists, otorhinolaryngologists, physiotherapists, and psychologists."<br>Emphasis on the importance of a prompt multi-disciplinary approach.
CASE REPORT: Dog Bites in Infancy, Trauma and Personality Development	1982	IL Gislason<br>JD Call	Case series of children bitten by dogs.<br>Retrospective.<br>Interviewed accompanied by parents on 4 separate occasions.<br>Semi structured interview.	N=3<br>M age 1<br>F age 2<br>F age 3	"(first child) personality changed from being charming and outgoing prior to the bite to being cautious and inhibited after the bite."<br>First child referring to dog as "the bad dog" and mentioning wanting to shoot the dog when he grows up.<br>Clinginess increased in second child after incident.<br>Second child had nightmares for 3 years.<br>Third child developed bed wetting.<br>Third child showed increased clinginess, thumb sucking, nightmares.<br>Third child described as "withdrawn" after incident.<br>All children more afraid of strange dogs after their incidents.<br>All mothers were psychologically shaken.
Use of contact desensitization and shaping in the treatment of Dog Phobia and Generalized Fear of the Outdoors	2010	SE Glasscock<br>WE MacLean Jr	Case study.<br>Prospective.<br>Combination of contact desensitization, shaping and family counselling.	N=1<br>F age 6	"subsequently developed a phobic reaction to dogs and an avoidance of playing outdoors" after dog bite.<br>Consistent response to DS and shaping.<br>Study showed evidence that treatment needed maintenance to avoid reverting back.
Microvascular replantation of a composite facial avulsion in a 24-month-child after dog bite	2017	D Macias<br>DI Kwon<br>PC Walker<br>NR Peterson	Case study.<br>Retrospective.	N=1<br>F age 2	8 months post-op "The patient's mood and activity level had returned to pre-trauma baseline according to the patient's mother."

Life-Threatening Panfacial Wild Dog Bites in a Child	2021	S Saha	Case study.<br>Retrospective.	N=1<br>M age 1	"Psychologically, for the first 6 mo, he experienced severe bouts of panic attacks from loud noises, especially the noises of lightning during storms."<br>"He improved thereafter, and after 9 mo, while still avoiding all dogs, he would sometimes be fearful after hearing the noise of leaves rustling during high winds."<br>"Otherwise, he recovered well under the supervision of a psychologist and a pediatrician."

Supplementary Table 2 – Descriptive studies (larger than n=4), and cross-sectional studies, using only retrospective investigation identified in searches of psychological consequences of dog bites to children.

**Paper title**	**Year**	**Author/s**	**Study design**	**Participants**	**Psychological findings**
Retrospective review of dog bite injuries in children presenting to a South Australian tertiary children's hospital emergency department	2014	SC Chiam<br>NS Solanki<br>M Lodge<br>M Higgins<br>AL Sparnon	Descriptive study.<br>Retrospective.<br>Review of children presenting to ED between 2009-2011.	N=277<br>Ages 0-17	2 children referred to a psychologist for management of post-traumatic stress.
Origins of fear of dogs in adults and children: The role of conditioning processes and prior familiarity with dogs	1991	S Doogan<br>GV Thomas	Analytical cross-sectional.<br>Retrospective.<br>Questionnaire to "investigate fear of dogs".<br>Adults and children asked to recall origin of their fear.<br>Using Geer's FSS-2.<br>Chi square testing for significance.	N=100 adults<br>N=30 children (8-9 years)	"No difference in the frequency of being bitten or chased reported by fearful vs non fearful groups".<br>"Significantly more fearful than non-fearful adults reported little contact with dogs prior to the onset of their fear (no significant difference in children)".
Facial dog bite injuries in children: Retrospective study of 77 cases	2012	B Hersant<br>S Cassier G<br>Constantinescu<br>P Gavelle<br>M -P Vazquez<br>A Picard<br>N Kadlub	Descriptive study.<br>Retrospective.<br>Study from 2002 to 2010.<br>"We analyzed epidemiological, clinical data, surgical outcomes".<br>Paper in French, only abstract available.	N=77<br>Mean age 5.36yrs.	35.1% of children had psychological problems afterward.
Pediatric Craniofacial Fractures from Canine Bites	2021	B Parent<br>MR Bykowski<br>FP Marji<br>S Ramgopal<br>JA Goldstein<br>JE Losee	Descriptive study.<br>Retrospective.<br>Included all pediatric dog bites between 2008-2019 at a single centre.<br>Selected cases with craniofacial fractures and described.	N=38 cases with craniofacial fractures described, of 3,602 paediatric dog bite encounters identified.	Four patients (11%) subsequently diagnosed with PTSD, characterized mainly by recurring nightmares.<br>Two of these patients have required extensive counselling and anxiolytic medications.

				Age and gender for this group not stated.	
Antecedents and consequences of pediatric dog bite injuries and their developmental trends: 101 cases in rural China	2014	J Shen<br>S Li<br>H Xiang<br>S Lu<br>D C Schwebel	Descriptive study.<br>Retrospective.<br>All families had a dog bite victim under 18Y in the last 12 months.<br>Structured interview with caregiver.	N=101<br>65% Male<br>Mean age 8.3 yrs	Children were reported to be generally unwilling to approach dogs after the incident and children tended to fear dogs to some degree after the incident.

Supplementary Table 3 – Descriptive studies (larger than n=4), and cross-sectional studies, using prospective follow-up, identified in searches of psychological consequences of dog bites to children.

**Paper title**	**Year**	**Author/s**	**Study design**	**Participants**	**Psychological findings**
Pediatric Dog Bite Victims: A Need for a Continuum of Care	2012	B Boat<br>C Dixon<br>E Pearl<br>L Thieken<br>SE Bucher	Descriptive study.<br>Prospective.<br>Inclusion criteria were 0Y-16Y with presenting complaint of "dog bite" to pediatric emergency department.<br>Parent completed a questionnaire after enrolment.<br>4 weeks later parents had 30 min phone interview.<br>Information obtained included: a) bite incident specifics, b) further medical/psychological care needed by child c) contact with additional agencies d) changes in the child's behaviour e) changes in parental concerns f) parent feedback on additional services	N=34<br>50 enrolled, 34 completed follow-up<br>Mean age 8yrs,<br>21 M, 13 F	No patients were hospitalised.<br>70% of victims demonstrated concerning behaviours since incident (most commonly talking a lot about the incident (29%), being fearful of dogs (27%), avoiding dogs (27%), being anxious or worried about seeing doctors or going to hospital (24%), being fearful (24%), having nightmares (21%).<br>Parents also reported concerns (eg. feeling guilty (59%), worried about scars (59), fearful for child's safety (44%).<br>No children received any psychological services.<br>50% of parents believed their children would benefit from interventions for help with fears.
Epidemiology of dog bites: A Belgian experience of canine behaviour and public health concerns	2006	T De Keuster<br>J Lamoureux<br>A Kahn	3 studies conducted in Belgium on bites in children, of which one relevant to our review.<br>Descriptive study.<br>Prospective.<br>Questionnaire delivered to parents of child victims of dog bites, 2-9 months after minor surgical treatment.	Study 3 N=22<br>Median age 7.5 (range 1-14 years).<br>12 M,10 F	"Among the 22 children, 12 had shown symptoms of PTSD for more than one month; 5 had all DSM-IV criteria for PTSD and 7 had some but not all criteria".<br>Symptoms included vivid recollection and re-living, avoidance behaviours or numbing, increased arousal, hypervigilance, nightmares, difficulties sleeping, aggression to siblings and peers.<br>No difference was found for presence of PTSD symptoms in regard to age, gender, time since the bite, type of dog, place of the accident, owner of the dog, childs activity at the time of the accident, or part of the child's body bitten.

					"Only the children who suffered severe and/or multiple bites developed full PTSD symptoms".
Investigation of Posttraumatic Stress Disorder in Children after Animal-Induced Injury in China	2010	L Ji<br>Z Xiaowei<br>W Chuanlin<br>L Wei	Descriptive study.<br>Prospective.<br>Reviewed 358 charts and follow up data.<br>"Family Apgar Scale assessment and PTSD screening were performed at ED admission".<br>"On week 1 after ED admission the patient underwent evaluation for acute stress disorder diagnosis by using the Child Acute Stress Questionnaire".<br>PTSD screening after 3M.	N=358<br>201 M<br>157 F<br>Mean age 9.4 yrs	38 met symptom criteria for ASD(Acute Stress Disorder).<br>19 patients developed PTSD – 10 of which had major injuries.<br>No significant differences regarding gender/age.<br>ASD early predictor of PTSD.
Child victims of dog bites treated in emergency departments: a prospective study	2003	A Kahn<br>P Bauche<br>J Lamoureux	Descriptive study.<br>Prospective.<br>Children with dog bites from 6 Eds.<br>Pediatricians and nurses collected standardized information via questionnaire.	N=100<br>Mean age 7.3 yrs<br>57 M, 43 F	Two children were treated by a psychologist: one child bitten on the face at home, the other bitten on the leg in a public place and left alone without care.
Assessment of 16-month Sequelae Due to Dog Bites Originally Studied in a French Multicenter Survey from 2009 to 2011	2018	G Pedrono<br>C Ricard<br>M Bouilly<br>C Beata<br>G Sarcey<br>B Thelot	Descriptive study.<br>Prospective.<br>485 dog bites at 8 hospital EDs, followed up 16 M later (Sep 2010-Dec 2011).<br>Data collected by telephone or email	N=298<br>Mean age 28.1 yrs, 42% <15yrs	Adult and children findings not reported separately, however:<br>47% reported long term consequences, 16% of these being psychological<br>1/7 still experienced pain 16M after bite.<br>27% reported morale effected, higher for F (32%) than males (22%).<br><32% respondents reported being afraid of dogs after being bitten, slightly less frequent with children <15Y.
Posttraumatic Stress Disorder After Dog Bites in Children	2003	V Peters<br>M Sottiaux<br>J Appleboom<br>A Kahn	Descriptive study.<br>Prospective.<br>Between Apr 2001 – Feb 2002.<br>Child dog bite victims at pediatric ED .	N=22<br>12 M, 10 F<br>Median age 7.5 yr	12/22 had PTSD symptoms for more than 1M, 5 had all DSM-IV criteria for PTSD; 7 children had some but not all criteria.<br>Symptoms included "vivid reviviscence of the traumatic event (12 children), avoidance behavior or

			Children under 16Y who received minor surgical treatment less than 48H after dog bite.<br>Parents agreed to do questionnaire 2-9M after bite.<br>Questionnaire with 60 items.<br>"Semistructured comprehensive interviews" by telephone.<br>8 questionnaires completed in further home visit.<br>"Statistical analyses were based on nonparametric tests and x^2 tests with Yates correction for small series"		numbing (7 children), and/or signs of increased arousal (6 children)".<br>"Only the children who had severe and/or multiple bites had full PTSD symptoms; none of the children with an accidental bite from their pet did so."<br>None received psychological support.
Symptomatology and Adaptive Functioning for Children Exposed to Normative Stressors, Dog Attack and Parental Violence	1997	BBR Rossman<br>RD Bingham<br>RN Emde	Descriptive study.<br>Prospective.<br>86 children aged 4-9 "interviewed after 3 types of aversive experience: repetitive exposure to parental violence, a dog attack, or mild stressor".<br>Parents completed (1) "family information and stressor structured interview", (2) "impact of event PTSD adult symptom questionnaire", (3) "PTSD Reaction Index", (4) "Child dissociative checklist", (5) "Life Events Questionnaire", (6) "Children's Behaviour Questionnaire", (7) "Child Behaviour Checklist".<br>Children completed (1) "structured stressor interview", (2) "PTSD reaction index", (3) "Child Dissociative Index", (4) "Peabody Picture Vocabulary Test".	N=14 dog attack (DA)<br>10M 4F<br>4-6yrs n=4<br>7-9yrs n=10	"Parent report PTSD symptoms greater in both the DA and PV groups.<br>Age and gender effects were significant for PTSD-Rl(p), showing greater symptoms for younger children and girls. However, interactions of group with age and gender showed that the overall age and gender trends occurred mainly in the DA group. In contrast, a group by gender interaction for child-reported PTSD symptoms and reflected in the DA group showed boys reporting more symptoms than girls. Interpretation of this result is complicated by the fact that although boys reported greater PTSD symptoms, parents observed girls to show higher symptom levels.<br>Mothers observed girls to have greater PTSD symptoms and boys to have greater dissociative symptoms, while child report showed boys to have greater PTSD symptoms.<br>Mothers' PTSD symptoms significantly or marginally predicted greater child internalising behaviour problems and lower social competence."

Psychological Adjustment in Children after Traumatic Disfiguring Injuries: A 12-Month Follow-Up	2000	MD Rusch,<br>BK Grunert<br>JR Sanger<br>WW Dzwierzynski<br>H Matloub	Descriptive study.<br>Prospective.<br>Children treated in plastic and reconstructive surgery department for traumatic facial or extremity injuries, which included dog bites.<br>Psychological evaluations performed within 4 days of injury.<br>Interviews for children and parents (structured).<br>Follow ups done at 1, 3, 6 and 12 month intervals.<br>Categories assessed: reexperiencing symptoms, avoidance of thoughts/conversations promoting memory of incident, impaired attention/concentration, reaction to disfigurement, phantom pain.	N=57<br>Age 3-12 yrs<br>40 M, 17 F<br>Not known which children suffered dog bites.	"Within 5 days of the traumatic event, 98 percent of the children were symptomatic for post traumatic stress disorder, depression or anxiety" (all but one child).<br>"One month after the injury, 82 percent were symptomatic".<br>"44 percent of the children continued to report symptoms at 12-month follow-up-visits, and 21 percent met the diagnostic criteria for posttraumatic stress disorder".<br>37% children reported fear of dying.<br>75% children reported fear of re-injury.<br>Children still experiencing fear of re-injury after 12M reduced partaking in "risky" or highly physical behaviours.<br>No statistical significance in age.<br>"82% of 17 girls (n=14) reported flashbacks at their 1-month follow up visits, compared with 45% of the 40 boys (n=18)(p=0.018). At the 12M follow up visits these differences no longer existed".<br>"44% of our subjects exhibited significant long term psychological symptoms after their injuries that were directly attributable to their accidental injuries".
Analysis of Dog Bites in Children Who Are Younger Than 17 Years	2006	J Schalamon<br>H Ainoedhofer<br>G Singer<br>T Petnehazy<br>J Mayr<br>K Kiss<br>ME Hollwarth	Descriptive study.<br>Retrospective/prospective.<br>Review of medical charts of children under 17Y who sought medical attention for a dog bite between 1994-2003 at pediatric surgery department.<br>Follow-up of 93% of the children 2-11 years after the dog attack (mean 7.2 years).	N=341<br>174 M, 167 F<br>Mean age 5.9yrs.	5 children complaining of nightmares after incidents.<br>Fear of dogs remained in 34 children during long-term follow-up.
Epidemiology and classification of dog bite injuries to the face: A prospective study of 108 patients	2015	G Toure<br>G Angoulangouli<br>J-P Meningaud	Descriptive study.<br>Prospective.<br>Study carried out over 13 years of dog bite intake to emergency surgeries.<br>Information sheet filled out by victims bitten on the face.	108 incidents of dog bites (to face and neck) out of 13021 patients<br>49 M<br>59 F	23% encountered complications, of which 20% were psychological shock (anxiety and post-traumatic stress, sleep disorder, phobia of dogs and the patients own image).

				69% <16yrs	
