# Supplementary material for: Review of psychological effects of dog bites in children
Source: BMJ Paediatr Open. 2024 Jun 6;8(1):e000922. doi: 10.1136/bmjpo-2020-000922 (PMC11163822; doi:10.1136/bmjpo-2020-000922)
Supplement: Author's manuscript [file bmjpo-2020-000922.draft_revisions.pdf]

BMJ Paediatrics Open

BMJ Paediatrics Open is committed to open peer review. As part of this commitment we make the peer review history of every article we publish publicly available.

When an article is published we post the peer reviewers' comments and the authors' responses online. We also post the versions of the paper that were used during peer review. These are the versions that the peer review comments apply to.

The versions of the paper that follow are the versions that were submitted during the peer review process. They are not the versions of record or the final published versions. They should not be cited or distributed as the published version of this manuscript.

BMJ Paediatrics Open is an open access journal and the full, final, typeset and author-corrected version of record of the manuscript is available on our site with no access controls, subscription charges or pay-per-view fees (http://bmjpaedsopen.bmj.com).

If you have any questions on BMJ Paediatrics Open's open peer review process please email info.bmjpo@bmj.com

BMJ Paediatrics Open

Psychological effects of dog bites in children

Journal:	*BMJ Paediatrics Open*
Manuscript ID	bmjpo-2020-000922
Article Type:	Review
Date Submitted by the Author:	02-Apr-2024
Complete List of Authors:	Westgarth, Carri; University of Liverpool Faculty of Health and Life Sciences, Department of Livestock and One Health; University of Liverpool<br>Provazza, Serena; Alder Hey Children's NHS Foundation Trust, Department of Clinical Health Psychology<br>Nicholas, Jade; Independent Researcher<br>Gray, Victoria; Alder Hey Children’s Hospital NHS Foundation Trust, Department of Clinical health Psychology
Keywords:	Child Health, Adolescent Health, Psychology

SCHOLARONE™
Manuscripts

BMJ

I, the Submitting Author has the right to grant and does grant on behalf of all authors of the Work (as defined in the below author licence), an exclusive licence and/or a non-exclusive licence for contributions from authors who are: i) UK Crown employees; ii) where BMJ has agreed a CC-BY licence shall apply, and/or iii) in accordance with the terms applicable for US Federal Government officers or employees acting as part of their official duties; on a worldwide, perpetual, irrevocable, royalty-free basis to BMJ Publishing Group Ltd ("BMJ") its licensees and where the relevant Journal is co-owned by BMJ to the co-owners of the Journal, to publish the Work in this journal and any other BMJ products and to exploit all rights, as set out in our licence.

The Submitting Author accepts and understands that any supply made under these terms is made by BMJ to the Submitting Author unless you are acting as an employee on behalf of your employer or a postgraduate student of an affiliated institution which is paying any applicable article publishing charge ("APC") for Open Access articles. Where the Submitting Author wishes to make the Work available on an Open Access basis (and intends to pay the relevant APC), the terms of reuse of such Open Access shall be governed by a Creative Commons licence – details of these licences and which Creative Commons licence will apply to this Work are set out in our licence referred to above.

Other than as permitted in any relevant BMJ Author's Self Archiving Policies, I confirm this Work has not been accepted for publication elsewhere, is not being considered for publication elsewhere and does not duplicate material already published. I confirm all authors consent to publication of this Work and authorise the granting of this licence.

Confidential: For Review Only

Psychological effects of dog bites in children

Westgarth, C[1]., Provazza, S[2]., Nicholas, J.[3], Gray, V[2].

1 Department of Livestock and One Health, Institute of Infection, Veterinary and Ecological Sciences, University of Liverpool, Leahurst Campus, Neston, Cheshire, CH66 1QU, UK

2 Department of Clinical Health Psychology, Alder Hey Children's NHS Foundation Trust, Eaton Road,Liverpool, Merseyside, L12 2AP, UK.

3 Independent Researcher, Hampshire, UK.

ABSTRACT

Dog bites are a concerning heath problem in children and one of the leading causes of non-fatal injuries in this population. Dog attacks not only cause physical injuries but can also lead to long-term psychological problems. A systematic review was performed to investigate the scope of literature on psychological effects of dog bites on a paediatric population. A literature search was performed on Web of Knowledge database between 1982-June 2023, returning 249 results. 14 primary studies reporting the psychological consequences of dog bites in children or adolescents were classed as eligible and 9 further studies added from prior knowledge and bibliographical searches. 23 studies involving 1894 participants met the criteria and were included in this review. Of these 23 studies, 8 were case studies or small case series reports (up to n=4), 14 larger descriptive studies, and 1 analytical cross-sectional study. There was a mixture of retrospective and prospective data-gathering. The most common psychological consequences of dog bites in children were post-traumatic stress disorder, dog phobia, nightmares and symptoms of anxiety and avoidance behaviours. Studies on dog bites in a paediatric population with a specific focus on the psychological consequeces associated with dog bites and their management are sparse. Future research and practice should more greatly consider the psychological impact in child victims of dog bites and their family members, as well as their management to avoid development of mental health

Confidential: For Review Only

issues and improve their quality of life. Future research also needs to ascertain the efficacy of using Virtual Reality in treating children with dog phobia.

KEY MESSAGES

- Psychological consequences of dog bites to children are common but under-researched and often overlooked.
- Typical consequences of dog bites are post-traumatic stress disorder, dog phobia, nightmares and symptoms of anxiety and avoidance behaviours.
- Treatment of dog phobias can be challenging to deliver.
- Virtual reality offers a potential ethical and safe, controlled environment for cognitive behavioural therapy regarding dogs.

INTRODUCTION

Dog ownership is argued to provide some health benefits [1-3], however, dogs, as well as other animals, may cause severe injuries to humans. Although there are no global estimates of dog bites incidence, the World Health Organization reports dog bites account for tens of millions of injuries annually [4]. As a result, dog bites represent a major public health concern, affecting predominately the paediatric population [5]. In the UK, incidence of hospital admissions for dog bites in children (14 or under) is stable at approximately 14.4 per 100,000 population per year [5].

It is well established that most of the child dog bite victims are bitten inside the home, and most dogs involved are known to the victim [6 7]. The COVID-19 global pandemic and the subsequent implementation of lockdowns with a 'stay at home' order forced children to spend more time than usual at home with a significant increase in dog bites [8 9].

Confidential: For Review Only

Some evidence supports that children are more vulnerable to dog attacks compared to adults because of their smaller physical size, their underestimation of risk and their tendency to behave more impulsively [10], as well as leaning-in behaviour with animals in young children [11]. Rates of admission peak in the 5-9 age group [8]. Furthermore, children are often injured in the neck and head regions and many have facial wounds [12-14]. The younger the child the more frequent the bites are to the face [14 15] and young children have more extensive/severe injuries [13 16].

In addition to physical impact, dog bites often carry health care costs [8]. They also produce psychological costs to the victims and other parties involved [17-19], however, compared to physical, psychological consequences of dog bites and their management is still scarcely investigated and poorly reported [20 21] Timely recognition of psychological symptoms caused by dog attacks in children and subsequent psychological support to the child victims and their families would be of critical importance to prevent development of future mental health issues, which would in turn influence the quality of life of the children and their caretakers. The aim of this systematic review was to explore current evidence on the psychological impact of dog bites in children and adolescents.

METHODS

Protocol and inclusion criteria

This systematic review was conducted according to the standards established by the Preferred Reporting Items for Systematic Reviews [22].

A search of Web of Knowledge database was performed to identify original research articles focusing on psychological consequences of dog bite injuries in the paediatric population from 1982-June 2023. The search term used on all fields was:

Confidential: For Review Only

("child* OR pediatric") AND (dog* OR cani*) AND (bit* OR attack*) AND (psychological OR PTSD OR phobia OR trauma* OR anxiety)

At least the abstract needed to be available in English for assessment for relevant inclusion, and screening was performed by CW and SP. Articles were excluded if: they were not an original primary source of evidence in a scientific journal (ie book chapter or review papers were excluded); the study population did not include children (ie under 18 years of age, although it could also include adults); studies focused on bites in general (e.g., human bites, cat bites etc) rather than dog; the information reported was not specific to psychological effects of dog bites but rather on dog bites in general (e.g., wound management, dog bite incidence etc). Articles were excluded based on reading the full paper if the title or abstract did not already clearly exclude it; if the study purpose and population was deemed relevant, often the full paper needed to be read in order to find if psychological effects had been reported. Further relevant studies were also identified from bibliographic searches and prior knowledge. See Figure 1 for study search and inclusion flowchart. Due to the low number of studies that met the inclusion criteria and low-level of evidence study designs used (case studies and descriptive case series), all studies have been reported here rather than exclusion based on quality. Evaluation and summary of the study design and psychological findings was performed by CW and JN.

RESULTS

Out of 249 papers screened for eligibility, 14 studies met the criteria and were included in this review, along with 9 other studies identified through prior knowledge and bioblographies, totalling 23 studies involving 1894 participants (Figure 1). Of these 23 studies, eight were single case studies or small case series (up to 4 children per report) [23-30] (Table 1). Thirteen were descriptive studies of larger patient groups [7 15 18 19 31-39], one was a case-control study but the psychological findings were only descriptional [40], and one was an analytical cross-sectional study [41] (Table 2). A mixture of

https://mc.manuscriptcentral.com/bmjpo

Confidential: For Review Only

retrospective (ie incidental review of past case records, survey regarding fear of dogs) and prospective data collection (deliberate interview of dog bite cases as they attended hospital, follow-up interview of a cohort of bitten children) methods were reported depending on the study, and sometimes both.

Summary of the findings

One study specific to dog bite patients estimated that 70% of children had demonstrated concerning behaviours since the incident [37]. Post-traumatic Stress Disorder was commonly encountered, being mentioned in eight studies: 2 of 4 children (a family in which one child was bitten) [23], diagnosed in a case study [28], diagnosed in descriptive studies of dog bite patients at a prevalence of 12/22 [31], 12/22 [19], 19/358 [18]; 98% within 5 days of a traumatic facial incident some of which were dog bites [35], and also mentioned more generally in two other studies, one which included (16%) child dog bite patients [34] and one specifically of child dog bites [36]. Acute Stress Disorder (ASD) was also mentioned (38/358 patients in [18]) and observed to be an early indicator of PTSD. In a study of children interviewed after a traumatic experience (16% of which were dog bites), PTSD symptoms were greater in the dog attack and parental violence groups than the 'mild stressor' group, and mothers of bitten children were also observed to develop PTSD which was associated with child PTSD score as reported by the parent [34]. In addition, in the dog attack group mothers observed girls to have greater PTSD symptoms and boys to have greater dissociative symptoms, while child self-report showed boys to have greater PTSD symptoms. Other studies did not find evidence of gender/age effects in regards to PTSD in child dog bites [18 31] The severity of the symptoms seemed to be related to the severity of the attacks; children who sustained more physically severe or multiple bites were more likely to develop PTSD symptoms [18 19 31].

Other than formally reporting PTSD/ASD, specific symptoms most commonly described in the articles were fearfulness/avoidance of dogs [24 25 27 30 33 36-39] and sleep disturbance/nightmares [23-25 31]

Confidential: For Review Only

[36-38 40]. Other symptoms included bed wetting [23 25], selective mutism [28], traumatic memories/re-living [19 28 31], talking a lot about the incident [37], increased arousal [19 31], anger/aggression [24 31], withdrawn/depressed/numbing [19 28 31 35], fearful [37], panic attacks [27], anxiety [35 36], hypervigilance [31], difficulty playing/avoidance of playing outdoors [24 30], anxiety about hospitals [37], fear of dying or re-injury [35], and phobia of own image [36].

Notably, parents reported changes in their behaviours as well, proving that dog bites represent a burden not only for the victims but also for their family [34 37] Parents described feeling shaken [25], guilty, fearful for child's safety and worried about scars [37].

Despite the reporting of psychological symptoms after dog bites in children, it was rare for psychological treatment to be recorded; although it featured in case studies [23 25 27-30], when evaluating descriptive studies it was only reported in 2/100 [15], 2/277 [7], 2/38 [40] patients. Indeed, two studies specifically noted no children received any psychological services (0/22 [19]; 0/34 [37]) but 50% of parents felt it would be helpful [37]. Hon et al (2007) acknowledged that one challenge to collecting these data is that in an emergency department service where dog bites present is it might be difficult to gather information about psychological and emotional trauma [21]. Clearly there is an unidentified gap in both the literature and patient need for psychological assessment. In addition it has been suggested that interventions should also include educational programmes on the risk and severity of dog attacks offered to parents and children, given dog bites can have significant psychological consequences that can negatively impact on children quality of life [29].

Confidential: For Review Only

DISCUSSION

The main purpose of this review was to explore the current literature on paediatric dog bites, with a specific focus on the psychological impacts of dog bites in children, as the psychological consequences of dog bites as well as their treatment are often overlooked [20 21]. Evidence suggested

that children bitten by dogs are at high risk of developing psychological disturbances, ranging from fear of dogs to clinical PTSD, however, few studies mentioned psychological management or treatment offered after dog attacks. It appears that assessing children in an emergency department (ED) to evaluate psychological sequelae following dog bites, and further offering them psychological support, can be difficult and/or lacks consideration. This may result in psychological symptoms significantly impacting children and potentially worsening. A form of follow-up assessment is likely to be required to assess whether initial symptoms of psychological distress have settled, but whom this responsibility falls to once a patient is discharged from the ED, is unclear. Further, not only did dog injuries have an impact on the victims, but they also affected their carers. Psychological symptoms showed by young victims of dog bites seemed to be long lasting with some studies reporting over 12 months. Age and gender differences were inconclusive between studies but across studies more severe bites commonly resulted in more serious psychological impact.

It is clear that many children who have been bitten by dogs or are otherwise fearful of dogs may benefit from psychological treatment. Although different approaches have been found to be effective in treating phobia for dogs in children, CBT (cognitive behavioural therapy) regarding some form of graded exposure appears to be the most widely utilised [42 43]. A difficulty with this is the ethical and practical safety implications regarding controlled exposure to real animals who can be difficult to access, unpredictable and to some extent uncontrollable in their behaviours, and with their own welfare considerations [44]. For example, one intervention states "*Parents were not provided with explicit instructions on how to find dogs but were encouraged to consider various options: dogs owned by extended family members, neighbors, and friends; dogs at pet stores, breeders, and animal shelters; dogs at the park or other recreation areas*" [43]. Another play-based intervention used dogs with handlers within the intensive session, with no description about how these had been assessed for suitability, and "*during the session, the child was encouraged to engage*

Confidential: For Review Only

in a range of different tasks with each dog including approaching and patting the dog, offering the dog treats and walking the dog on the lead" [42]. Great care must be taken in order to be able to gradually control the intensity of exposure and also safeguard both child and animal welfare during such treatment so that neither becomes overwhelmed and both have a positive experience. This includes providing very clear guidance to those delivering it, including parents. A potential more ethical solution to these challenges during the intensive and early stages of therapy would be to use virtual reality [44]. This would allow a gradual and controlled increase in exposure to particular dog behaviours required for that case. For example, Farrel (2021) have suggested the use of Virtual Reality therapy in treating dog phobia can be possible using just one treatment session [45]. In eight children with specific phobia of dogs, after one month follow-up, 75% of the children were considered recovered. The authors concluded that VR can be effectively be used as an alternative to the classic in-vivo exposure-based therapy and might overcome some of its challenges as the most difficult CBT technique to deliver. More research is needed to confirm the efficacy of VR as an effective treatment of dog fear in a paediatric population.

This review has the merit of investigating and summarising existing evidence on psychological effects of paediatric dog bites and their management, as to the best of our knowledge, such investigation has not been conducted before. However, it presents with some limitations. First of all, even though the literature search was performed on a highly relevant database, some studies may be missing from this review. Additionally, of the few studies identified, many had a very limited sample size, which may limited the statistical power and generalizability of the findings. Therefore, the conclusions drawn here should be taken with caution. Notwithstanding the above limitations, this review provides an insight into the psychological consequences of paediatric dog bites.

https://mc.manuscriptcentral.com/bmjpo

Confidential: For Review Only

To conclude, dog bites in children represent a traumatic event, which can cause devasting psychological consequences in the victims and in their families. A thorough investigation of the psychological impact of dog bites on children and their parents followed by a prompt multidisciplinary management of both physical and psychological symptoms would lead to better outcomes, preventing the occurrence of more severe mental health problems such as phobia and PTSD and improve the quality of life of the victims and their families. Furthermore, educational programmes on the risk and severity of dog attacks should be offered to parents and children, to prevent dog bites. Cognitive behavioural therapy can be used to treat fear of dogs in children, with VR representing an alternative to the classic in vivo exposition therapy. Future research should focus more on the psychological impact of dog injuries and on the treatment of child victims of dog bites and their family members, to avoid development of mental health issues and improve their quality of life.

FUNDING

This work was supported by the Faculty of Health and Life Sciences, University of Liverpool.

COMPETING INTERESTS

CW is a member of the Merseyside Dog Safety Partnership, which has a website containing useful resources for dog bite prevention. www.merseydogsafe.co.uk. There are no other competing interests to declare.

RESEARCH ETHICS APPROVAL

Not applicable - this study does not involve human participants.

CONTRIBUTORS

CW conceptualised the paper. CW and SP performed the searches and paper selection. CW and JN reviewed the papers and assimilated the data. CW and SP wrote the first draft. All authors commented on and revised the paper. CW submitted the paper.

DATA AVAILABILITY

Confidential: For Review Only

No other datasets were generated.

PATIENT CONSENT FOR PUBLICATION

Not applicable.

PROVENANCE AND PEER REVIEW

Commissioned; externally peer reviewed.

Confidential: For Review Only

REFERENCES

1. Herzog H. The Impact of Pets on Human Health and Psychological Well-Being: Facto, Fiction, or Hypothesis? *Current Directions in Psychological Science* 2011;20:236-39.
2. O'Haire M. Companion animals and human health: Benefits, challenges, and the road ahead. *Journal of Veterinary Behaviour* 2010;5:226-34.
3. Rodriguez KE, Herzog H, Gee NR. Variability in Human-Animal Interaction Research. *Front Vet Sci* 2021;7:9. doi: 10.3389/fvets.2020.619600
4. WHO. Animal Bites: World Health Organisation; 2018 [cited 2024 9th January]. Available from: https://www.who.int/news-room/fact-sheets/detail/animal-bites.
5. Tulloch JSP, Owczarczak-Garstecka SC, Fleming KM, et al. English hospital episode data analysis (1998–2018) reveal that the rise in dog bite hospital admissions is driven by adult cases. *Scientific Reports* 2021;11(1):1767. doi: 10.1038/s41598-021-81527-7
6. Cornelissen JMR, Hopster H. Dog bites in The Netherlands: A study of victims, injuries, circumstances and aggressors to support evaluation of breed specific legislation. *Veterinary Journal* 2010;186(3):292-98. doi: 10.1016/j.tvjl.2009.10.001
7. Chiam SC, Solanki NS, Lodge M, et al. Retrospective review of dog bite injuries in children presenting to a South Australian tertiary children's hospital emergency department. *J Paediatr Child Health* 2014;50(10):791-4. doi: 10.1111/jpc.12642 [published Online First: 2014/07/22]
8. Tulloch JSP, Minford S, Pimblett V, et al. Paediatric emergency department dog bite attendance during the COVID-19 pandemic: an audit at a tertiary children's hospital. *BMJ paediatrics open* 2021;5(1):e001040-e40. doi: 10.1136/bmjpo-2021-001040
9. Parente G, Gargano T, Di Mitri M, et al. Consequences of COVID-19 Lockdown on Children and Their Pets: Dangerous Increase of Dog Bites among the Paediatric Population. *Children (Basel)* 2021;8(8) doi: 10.3390/children8080620 [published Online First: 2021/08/28]
10. Shen J, Rouse J, Godbole M, et al. Systematic Review: Interventions to Educate Children About Dog Safety and Prevent Pediatric Dog-Bite Injuries: A Meta-Analytic Review. *J Pediatr Psychol* 2017;42(7):779-91. doi: 10.1093/jpepsy/jsv164
11. Meints K, Syrnyk C, De Keuster T. Why do children get bitten in the face? *Injury Prevention* 2010;16(Suppl 1):A172-A73. doi: 10.1136/ip.2010.029215.617
12. Kasbekar AV, Garfit H, Duncan C, et al. Dog bites to the head and neck in children; an increasing problem in the UK. *Clinical Otolaryngology* 2013;38(3):259-62. doi: 10.1111/coa.12094
13. Khan K, Horswell BB, Samanta D. Dog-Bite Injuries to the Craniofacial Region: An Epidemiologic and Pattern-of-Injury Review at a Level 1 Trauma Center. *Journal of Oral and Maxillofacial Surgery* 2020;78(3):401-13. doi: https://doi.org/10.1016/j.joms.2019.11.002

14. Sribnick EA, Sarda S, Sawvel MS, et al. Dog bite injuries in children: Clinical implications for head involvement. *Trauma* 2016;18(3):210-15. doi: 10.1177/1460408616631772
15. Kahn A, Bauche P, Lamoureux J, et al. Child victims of dog bites treated in emergency departments: a prospective survey. *European Journal of Pediatrics* 2003;162(4):254-58. doi: 10.1007/s00431-002-1130-6
16. Brogan TV, Bratton SL, Dowd MD, et al. SEVERE DOG BITES IN CHILDREN. *Pediatrics* 1995;96(5):947-50.
17. Westgarth C, Watkins F. Chapter 23: Impact of dog aggression on victims. In: Mills DS, Westgarth C, eds. Dog Bites: A Multidisciplinary Perspective. 1st Edition ed. Sheffield: 5M Publishing 2017:309-20.
18. Ji L, Xiaowei Z, Chuanlin W, et al. Investigation of posttraumatic stress disorder in children after animal-induced injury in China. *Pediatrics* 2010;126(2):e320-4. doi: 10.1542/peds.2009-3530 [published Online First: 2010/07/14]
19. Peters V, Sottiaux M, Appelboom J, et al. Posttraumatic stress disorder after dog bites in children. *The Journal of Pediatrics* 2004;144(1):121-22. doi: http://dx.doi.org/10.1016/j.jpeds.2003.10.024
20. Jakeman M, Oxley JA, Owczarczak-Garstecka SC, et al. Pet dog bites in children: management and prevention. *BMJ Paediatrics Open* 2020;4(1):e000726. doi: 10.1136/bmjpo-2020-000726
21. Hon KLE, Fu CCA, Chor CM, et al. Issues associated with dog bite injuries in children and adolescents assessed at the emergency department. *Pediatric Emergency Care* 2007;23(7):445-49.
22. Page MJ, McKenzie JE, Bossuyt PM, et al. The PRISMA 2020 statement: an updated guideline for reporting systematic reviews. *BMJ* 2021;372:n71. doi: 10.1136/bmj.n71
23. Albano AM, Miller PP, Zarate R, et al. Behavioral assessment and treatment of PTSD in prepubertal children: Attention to developmental factors and innovative strategies in the case study of a family. *Cognitive and Behavioral Practice* 1997;4(2):245-62. doi: https://doi.org/10.1016/S1077-7229(97)80003-X
24. Drell MJ, Gaensbauer TJ, Siegel CH, et al. Clinical round table: A case of trauma to a 21-month-old girl. *Infant Mental Health Journal* 1995;16(4):318-33. doi: https://doi.org/10.1002/1097-0355(199524)16:4<318::AID-IMHJ2280160407>3.0.CO;2-L
25. Gislason IL, Call JD. Dog Bite in Infancy: Trauma and Personality Development. *Journal of the American Academy of Child Psychiatry* 1982;21(2):203-07. doi: https://doi.org/10.1016/S0002-7138(09)60921-3
26. Macias D, Kwon DI, Walker PC, et al. Microvascular replantation of a composite facial avulsion in a 24-month-old child after dog bite. *Microsurgery* 2018;38(2):218-21. doi: https://doi.org/10.1002/micr.30275
27. Saha S. Life-Threatening Panfacial Wild Dog Bites in a Child. *Wilderness & Environmental Medicine* 2021;32(4):511-16. doi: https://doi.org/10.1016/j.wem.2021.07.001
28. Anyfantakis D, Botzakis E, Mplevrakis E, et al. Selective mutism due to a dog bite trauma in a 4-year-old girl: A case report. *Journal of Medical Case Reports* 2009;3
29. de Carvalho MF, Hardtke LA, de Souza MF, et al. Comminuted mandibular fracture in child victim of dog bite. *Dent Traumatol* 2012;28(4):324-8. doi: 10.1111/j.1600-9657.2011.01079.x [published Online First: 2011/11/22]
30. Glasscock SE, MacLean Jr WE. Use of Contact Desensitization and Shaping in the Treatment of Dog Phobia and Generalized Fear of the Outdoors. *Journal of Clinical Child Psychology* 1990;19(2):169-71. doi: 10.1207/s15374424jccp1902_9
31. De Keuster T, Lamoureux J, Kahn A. Epidemiology of dog bites: A Belgian experience of canine behaviour and public health concerns. *Veterinary Journal* 2006;172(3):482-87.

Confidential: For Review Only

32. Hersant B, Cassier S, Constantinescu G, et al. Facial dog bite injuries in children: Retrospective study of 77 cases. *Annales De Chirurgie Plastique Esthetique* 2012;57(3):230-39. doi: 10.1016/j.anplas.2011.11.003
33. Pédrono G, Ricard C, Bouilly M, et al. Assessment of 16-month Sequelae Due to Dog Bites Originally Studied in a French Multicenter Survey from 2009 to 2011. *Wounds* 2018;30(5):84-89. [published Online First: 2018/03/10]
34. Rossman BR, Bingham RD, Emde RN. Symptomatology and adaptive functioning for children exposed to normative stressors, dog attack, and parental violence. *J Am Acad Child Adolesc Psychiatry* 1997;36(8):1089-97. doi: 10.1097/00004583-199708000-00016 [published Online First: 1997/08/01]
35. Rusch MD, Grunert BK, Sanger JR, et al. Psychological adjustment in children after traumatic disfiguring injuries: A 12-month follow-up. *Plastic and Reconstructive Surgery* 2000;106(7):1451-58.
36. Touré G, Angoulangouli G, Méningaud J-P. Epidemiology and classification of dog bite injuries to the face: A prospective study of 108 patients. *Journal of Plastic, Reconstructive & Aesthetic Surgery* 2015;68(5):654-58. doi: https://doi.org/10.1016/j.bjps.2015.01.001
37. Boat BW, Dixon CA, Pearl E, et al. Pediatric dog bite victims: a need for a continuum of care. *Clin Pediatr (Phila)* 2012;51(5):473-7. doi: 10.1177/0009922811435504 [published Online First: 2012/02/02]
38. Schalamon J, Ainoedhofer H, Singer G, et al. Analysis of dog bites in children who are younger than 17 years. *Pediatrics* 2006;117(3):E374-E79. doi: 10.1542/peds.2005-1451
39. Shen J, Li S, Xiang H, et al. Antecedents and consequences of pediatric dog-bite injuries and their developmental trends: 101 cases in rural China. *Accident Analysis and Prevention* 2014;63:22-29.
40. Parent B, Bykowski MR, Marji FP, et al. Pediatric Craniofacial Fractures From Canine Bites. *J Craniofac Surg* 2021;32(4):1627-32. doi: 10.1097/scs.0000000000007546 [published Online First: 2021/03/21]
41. Doogan S, Thomas GV. Origins of fear of dogs in adults and children: The role of conditioning processes and prior familiarity with dogs. *Behaviour Research and Therapy* 1992;30(4):387-94. doi: https://doi.org/10.1016/0005-7967(92)90050-Q
42. Farrell LJ, Kershaw H, Ollendick T. Play-Modified One-Session Treatment for Young Children with a Specific Phobia of Dogs: A Multiple Baseline Case Series. *Child Psychiatry Hum Dev* 2018;49(2):317-29. doi: 10.1007/s10578-017-0752-x [published Online First: 2017/08/03]
43. Radtke SR, Muskett A, Coffman MF, et al. Bibliotherapy for Specific Phobias of Dogs in Young Children: A Pilot Study. *Journal of Child and Family Studies* 2023;32(2):373-83. doi: 10.1007/s10826-022-02304-2
44. Oxley JA, Santa K, Meyer G, et al. A Systematic Scoping Review of Human-Dog Interactions in Virtual and Augmented Reality: The Use of Virtual Dog Models and Immersive Equipment. *Frontiers in Virtual Reality* 2022;3 doi: 10.3389/frvir.2022.782023
45. Farrell LJ, Miyamoto T, Donovan CL, et al. Virtual Reality One-Session Treatment of Child-Specific Phobia of Dogs: A Controlled, Multiple Baseline Case Series. *Behavior Therapy* 2021;52(2):478-91. doi: https://doi.org/10.1016/j.beth.2020.06.003

Confidential: For Review Only

FIGURES

Figure 1. Flow chart of study search and screening process for studies of psychological consequences of dog bites to children

Confidential: For Review Only

TABLES

Table 1: Case studies/small case-series identified in searches of psychological consequences of dog bites to children.

Paper title	Year	Author/s	Study design	Participants	Psychological findings
Behavioural Assessment and Treatment of PTSD in Prepubertal Children: Attention to Developmental Factors and Innovative Strategies in the Case Study of a Family	1997	AM Albano<br>PP Miller<br>G CotéDH Barlow	Mixed methods case study observing one family after a traumatic incident dog bite.<br>Prospective.<br>Diagnostic interviews.<br>Observational methods including rating sleep cycles of children, recording enuresis episodes and recording incidents of new behaviour since the incident.<br>Family behavioural tests with units of distress calculated.	N=4<br>M age 7<br>F age 6 (bitten)<br>F age 4<br>F age 2	Oldest 2 children diagnosed with PTSD.<br>Nocturnal changes in 2 children (bed wetting) represents a "significant change in functioning".<br>All 4 children experienced moderate to severe sleep disturbances.<br>"It was noted that as the age of the child increased, the more cognitively mediated symptoms and expression of fear became apparent."<br>Adult symptoms went into remission without treatment.
Selective mutism due to a dog bite trauma in a 4-year-old girl: a case report	2009	D Anyfantakis<br>E Botzakis<br>E Mplevrakis<br>EK Symvoulakis<br>I Arbiros	Case study.<br>Retrospective.	N=1<br>F age 5	On admission was confused and lethargic – findings showed hypovolemic shock.<br>2nd day of hospitalisation – child was withdrawn and depressed – used non verbal communication.<br>2 months after discharge was non-verbal and had reccurent traumatic memories.<br>Case met all criteria for selective mutism and acute PTSD.<br>Treatment consisted of "supportive psychotherapy for the child and consecutive sessions of counselling for her parents".<br>Improvement after 6M.
Clinical Round Table: A Case of Trauma to a 21-Month-Old Girl	1995	MJ Drell<br>TJ Gaensbauer<br>CH Siegel<br>M Sugar	Case study.<br>Retrospective.	N=1<br>F age 1	Detailed account of symptoms and therapy of child and mother.<br>Child displayed fearfulness of dogs, nightmares, anger, difficulty playing.

Confidential: For Review Only

Comminuted mandibular fracture in child victim of dog bite	2012	M Furtado de Carvalho<br>LAP Hardtke<br>MF Cota de Souza<br>V de Oliveira Araujo	Case study.<br>Retrospective.	N=1<br>M age 3	The patient was seen every day by oral and maxillofacial surgeons, dieticians, pediatricians, plastic surgeons, ophthalmologists, otorhinolaryngologists, physiotherapists, and psychologists."<br>Emphasis on the importance of a prompt multi-disciplinary approach.
CASE REPORT: Dog Bites in Infancy, Trauma and Personality Development	1982	IL Gislason<br>JD Call	Case series of children bitten by dogs.<br>Retrospective.<br>Interviewed accompanied by parents on 4 separate occasions.<br>Semi structured interview.	N=3<br>M age 1<br>F age 2<br>F age 3	"(first child) personality changed from being charming and outgoing prior to the bite to being cautious and inhibited after the bite."<br>First child referring to dog as "the bad dog" and mentioning wanting to shoot the dog when he grows up.<br>Clinginess increased in second child after incident.<br>Second child had nightmares for 3 years.<br>Third child developed bed wetting.<br>Third child showed increased clinginess, thumb sucking, nightmares.<br>Third child described as "withdrawn" after incident.<br>All children more afraid of strange dogs after their incidents.<br>All mothers were psychologically shaken.
Use of contact desensitization and shaping in the treatment of Dog Phobia and Generalized Fear of the Outdoors	2010	SE Glasscock<br>WE MacLean Jr	Case study.<br>Prospective.<br>Combination of contact desensitization, shaping and family counselling.	N=1<br>F age 6	"subsequently developed a phobic reaction to dogs and an avoidance of playing outdoors" after dog bite.<br>Consistent response to DS and shaping.<br>Study showed evidence that treatment needed maintenance to avoid reverting back.
Microvascular replantation of a composite facial avulsion in a 24-month-child after dog bite	2017	D Macias<br>DI Kwon<br>PC Walker<br>NR Peterson	Case study.<br>Retrospective.	N=1<br>F age 2	8 months post-op "The patient's mood and activity level had returned to pre-trauma baseline according to the patient's mother."
Life-Threatening Panfacial Wild Dog Bites in a Child	2021	S Saha	Case study.<br>Retrospective.	N=1<br>M age 1	"Psychologically, for the first 6 mo, he experienced severe bouts of panic attacks from loud noises,

https://mc.manuscriptcentral.com/bmjpo

Confidential: For Review Only

					especially the noises of lightning during storms." "He improved thereafter, and after 9 mo, while still avoiding all dogs, he would sometimes be fearful after hearing the noise of leaves rustling during high winds." "Otherwise, he recovered well under the supervision of a psychologist and a pediatrician."

Table 2 – Descriptive studies (larger than n=4), and cross-sectional studies, using both retrospective investigation and prospective follow-up, identified in searches of psychological consequences of dog bites to children.

**Paper title**	**Year**	**Author/s**	**Study design**	**Participants**	**Psychological findings**
Pediatric Dog Bite Victims: A Need for a Continuum of Care	2012	B Boat<br>C Dixon<br>E Pearl<br>L Thieken<br>SE Bucher	Descriptive study.<br>Prospective.<br>Inclusion criteria were 0Y-16Y with presenting complaint of "dog bite" to pediatric emergency department.<br>Parent completed a questionnaire after enrolment.<br>4 weeks later parents had 30 min phone interview.<br>Information obtained included: a) bite incident specifics, b) further medical/psychological care needed by child c) contact with additional agencies d) changes in the child's behaviour e) changes in parental concerns f) parent feedback on additional services	N=34<br>50 enrolled, 34 completed follow-up<br>Mean age 8yrs,<br>21 M, 13 F	No patients were hospitalised.<br>70% of victims demonstrated concerning behaviours since incident (most commonly talking a lot about the incident (29%), being fearful of dogs (27%), avoiding dogs (27%), being anxious or worried about seeing doctors or going to hospital (24%), being fearful (24%), having nightmares (21%).<br>Parents also reported concerns (eg. feeling guilty (59%), worried about scars (59), fearful for child's safety (44%).<br>No children received any psychological services.<br>50% of parents believed their children would benefit from interventions for help with fears.
Retrospective review of dog bite injuries in children presenting to a South Australian tertiary children's hospital emergency department	2014	SC Chiam<br>NS Solanki<br>M Lodge<br>M Higgins<br>AL Sparnon	Descriptive study.<br>Retrospective.<br>Review of children presenting to ED between 2009-2011.	N=277<br>Ages 0-17	2 children referred to a psychologist for management of post-traumatic stress.
Epidemiology of dog bites: A Belgian experience of	2006	T De Keuster<br>J Lamoureux<br>A Kahn	3 studies conducted in Belgium on bites in children, of which one relevant to our review.	Study 3<br>N=22	"Among the 22 children, 12 had shown symptoms of PTSD for more than one month; 5 had all

Confidential: For Review Only

canine behaviour and public health concerns			Descriptive study. Prospective. Questionnaire delivered to parents of child victims of dog bites, 2-9 months after minor surgical treatment.	Median age 7.5 (range 1-14 years). 12 M,10 F	DSM-IV criteria for PTSD and 7 had some but not all criteria". Symptoms included vivid recollection and re-living, avoidance behaviours or numbing, increased arousal, hypervigilance, nightmares, difficulties sleeping, aggression to siblings and peers. No difference was found for presence of PTSD symptoms in regard to age, gender, time since the bite, type of dog, place of the accident, owner of the dog, childs activity at the time of the accident, or part of the child's body bitten. "Only the children who suffered severe and/or multiple bites developed full PTSD symptoms".
Origins of fear of dogs in adults and children: The role of conditioning processes and prior familiarity with dogs	1991	S Doogan GV Thomas	Analytical cross-sectional. Retrospective. Questionnaire to "investigate fear of dogs". Adults and children asked to recall origin of their fear. Using Geer's FSS-2. Chi square testing for significance.	N=100 adults N=30 children (8-9 years)	"No difference in the frequency of being bitten or chased reported by fearful vs non fearful groups". "Significantly more fearful than non-fearful adults reported little contact with dogs prior to the onset of their fear (no significant difference in children)".
Facial dog bite injuries in children: Retrospective study of 77 cases	2012	B Hersant S Cassier G Constantinescu P Gavelle M -P Vazquez A Picard N Kadlub	Descriptive study. Retrospective. Study from 2002 to 2010. "We analyzed epidemiological, clinical data, surgical outcomes". Paper in French, only abstract available.	N=77 Mean age 5.36yrs.	35.1% of children had psychological problems afterward.
Investigation of Posttraumatic Stress Disorder in Children after Animal-Induced Injury in China	2010	L Ji Z Xiaowei W Chuanlin L Wei	Descriptive study. Prospective. Reviewed 358 charts and follow up data. "Family Apgar Scale assessment and PTSD screening were performed at ED admission". "On week 1 after ED admission the patient underwent evaluation for acute stress disorder diagnosis by using the Child Acute Stress Questionnaire". PTSD screening after 3M.	N=358 201 M 157 F Mean age 9.4 yrs	38 met symptom criteria for ASD(Acute Stress Disorder). 19 patients developed PTSD – 10 of which had major injuries. No significant differences regarding gender/age. ASD early predictor of PTSD.

Confidential: For Review Only

Child victims of dog bites treated in emergency departments: a prospective study	2003	A Kahn<br>P Bauche<br>J Lamoureux	Descriptive study.<br>Prospective.<br>Children with dog bites from 6 Eds.<br>Pediatricians and nurses collected standardized information via questionnaire.	N=100<br>Mean age 7.3 yrs<br>57 M, 43 F	Two children were treated by a psychologist: one child bitten on the face at home, the other bitten on the leg in a public place and left alone without care.
Pediatric Craniofacial Fractures from Canine Bites	2021	B Parent<br>MR Bykowski<br>FP Marji<br>S Ramgopal<br>JA Goldstein<br>JE Losee	Descriptive study.<br>Retrospective.<br>Included all pediatric dog bites between 2008-2019 at a single centre.<br>Selected cases with craniofacial fractures and described.	N=38 cases with craniofacial fractures described, of 3,602 paediatric dog bite encounters identified.<br>Age and gender for this group not stated.	Four patients (11%) subsequently diagnosed with PTSD, characterized mainly by recurring nightmares.<br>Two of these patients have required extensive counselling and anxiolytic medications.
Assessment of 16-month Sequelae Due to Dog Bites Originally Studied in a French Multicenter Survey from 2009 to 2011	2018	G Pedrono<br>C Ricard<br>M Bouilly<br>C Beata<br>G Sarcey<br>B Thelot	Descriptive study.<br>Prospective.<br>485 dog bites at 8 hospital EDs, followed up 16 M later (Sep 2010-Dec 2011).<br>Data collected by telephone or email	N=298<br>Mean age 28.1 yrs,<br>42% <15yrs	Adult and children findings not reported separately, however:<br>47% reported long term consequences, 16% of these being psychological<br>1/7 still experienced pain 16M after bite.<br>27% reported morale effected, higher for F (32%) than males (22%).<br><32% respondents reported being afraid of dogs after being bitten, slightly less frequent with children <15Y.
Posttraumatic Stress Disorder After Dog Bites in Children	2003	V Peters<br>M Sottiaux<br>J Appleboom<br>A Kahn	Descriptive study.<br>Prospective.<br>Between Apr 2001 – Feb 2002.<br>Child dog bite victims at pediatric ED .<br>Children under 16Y who received minor surgical treatment less than 48H after dog bite.<br>Parents agreed to do questionnaire 2-9M after bite.<br>Questionnaire with 60 items.<br>“Semistructured comprehensive interviews” by telephone.<br>8 questionnaires completed in further home visit.<br>“Statistical analyses were based on nonparametric	N=22<br>12 M, 10 F<br>Median age 7.5 yr	12/22 had PTSD symptoms for more than 1M, 5 had all DSM-IV criteria for PTSD; 7 children had some but not all criteria.<br>Symptoms included “vivid reviviscence of the traumatic event (12 children), avoidance behavior or numbing (7 children), and/or signs of increased arousal (6 children)”.<br>“Only the children who had severe and/or multiple bites had full PTSD symptoms; none of the children with an accidental bite from their pet did so.”

Confidential: For Review Only

			tests and x^2 tests with Yates correction for small series"		None received psychological support.
Symptomatology and Adaptive Functioning for Children Exposed to Normative Stressors, Dog Attack and Parental Violence	1997	BBR Rossman<br>RD Bingham<br>RN Emde	Descriptive study.<br>Prospective.<br>86 children aged 4-9 "interviewed after 3 types of aversive experience: repetitive exposure to parental violence, a dog attack, or mild stressor".<br>Parents completed (1) "family information and stressor structured interview", (2) "impact of event PTSD adult symptom questionnaire", (3) "PTSD Reaction Index", (4) "Child dissociative checklist", (5) "Life Events Questionnaire", (6) "Children's Behaviour Questionnaire", (7) "Child Behaviour Checklist".<br>Children completed (1) "structured stressor interview", (2) "PTSD reaction index", (3) "Child Dissociative Index", (4) "Peabody Picture Vocabulary Test".	N=14 dog attack (DA)<br>10M 4F<br>4-6yrs n=4<br>7-9yrs n=10	"Parent report PTSD symptoms greater in both the DA and PV groups. Age and gender effects were significant for PTSD-Rl(p), showing greater symptoms for younger children and girls. However, interactions of group with age and gender showed that the overall age and gender trends occurred mainly in the DA group. In contrast, a group by gender interaction for child-reported PTSD symptoms and reflected in the DA group showed boys reporting more symptoms than girls. Interpretation of this result is complicated by the fact that although boys reported greater PTSD symptoms, parents observed girls to show higher symptom levels. Mothers observed girls to have greater PTSD symptoms and boys to have greater dissociative symptoms, while child report showed boys to have greater PTSD symptoms.<br>Mothers' PTSD symptoms significantly or marginally predicted greater child internalising behaviour problems and lower social competence."
Psychological Adjustment in Children after Traumatic Disfiguring Injuries: A 12-Month Follow-Up	2000	MD Rusch,<br>BK Grunert<br>JR Sanger<br>WW Dzwierzynski<br>H Matloub	Descriptive study.<br>Prospective.<br>Children treated in plastic and reconstructive surgery department for traumatic facial or extremity injuries, which included dog bites.<br>Psychological evaluations performed within 4 days of injury.<br>Interviews for children and parents (structured).<br>Follow ups done at 1, 3, 6 and 12 month intervals.	N=57<br>Age 3-12 yrs<br>40 M, 17 F<br>Not known which children suffered dog bites.	"Within 5 days of the traumatic event, 98 percent of the children were symptomatic for post traumatic stress disorder, depression or anxiety" (all but one child).<br>"One month after the injury, 82 percent were symptomatic".<br>"44 percent of the children continued to report symptoms at 12-month follow-up-visits,

1

			Categories assessed: reexperiencing symptoms, avoidance of thoughts/conversations promoting memory of incident, impaired attention/concentration, reaction to disfigurement, phantom pain.		and 21 percent met the diagnostic criteria for posttraumatic stress disorder". 37% children reported fear of dying. 75% children reported fear of re-injury. Children still experiencing fear of re-injury after 12M reduced partaking in "risky" or highly physical behaviours. No statistical significance in age. "82% of 17 girls (n=14) reported flashbacks at their 1-month follow up visits, compared with 45% of the 40 boys (n=18)(p=0.018). At the 12M follow up visits these differences no longer existed". "44% of our subjects exhibited significant long term psychological symptoms after their injuries that were directly attributable to their accidental injuries".
Analysis of Dog Bites in Children Who Are Younger Than 17 Years	2006	J Schalamon<br>H Ainoedhofer<br>G Singer<br>T Petnehazy<br>J Mayr<br>K Kiss<br>ME Hollwarth	Descriptive study. Retrospective/prospective. Review of medical charts of children under 17Y who sought medical attention for a dog bite between 1994-2003 at pediatric surgery department. Follow-up of 93% of the children 2-11 years after the dog attack (mean 7.2 years).	N=341<br>174 M, 167 F<br>Mean age 5.9yrs.	5 children complaining of nightmares after incidents. Fear of dogs remained in 34 children during long-term follow-up.
Antecedents and consequences of pediatric dog bite injuries and their developmental trends: 101 cases in rural China	2014	J Shen<br>S Li<br>H Xiang<br>S Lu<br>D C Schwebel	Descriptive study. Retrospective. All families had a dog bite victim under 18Y in the last 12 months. Structured interview with caregiver.	N=101<br>65% Male<br>Mean age 8.3 yrs	Children were reported to be generally unwilling to approach dogs after the incident and children tended to fear dogs to some degree after the incident.
Epidemiology and classification of dog bite injuries to the face: A prospective study of 108 patients	2015	G Toure<br>G Angoulangouli<br>J-P Meningaud	Descriptive study. Prospective. Study carried out over 13 years of dog bite intake to emergency surgeries. Information sheet filled out by victims bitten on the face.	108 incidents of dog bites (to face and neck) out of 13021 patients<br>49 M	23% encountered complications, of which 20% were psychological shock (anxiety and post-traumatic stress, sleep disorder, phobia of dogs and the patients own image).

Confidential: For Review Only

				59 F<br>69% <16yrs	

Confidential: For Review Only

Confidential: For Review Only

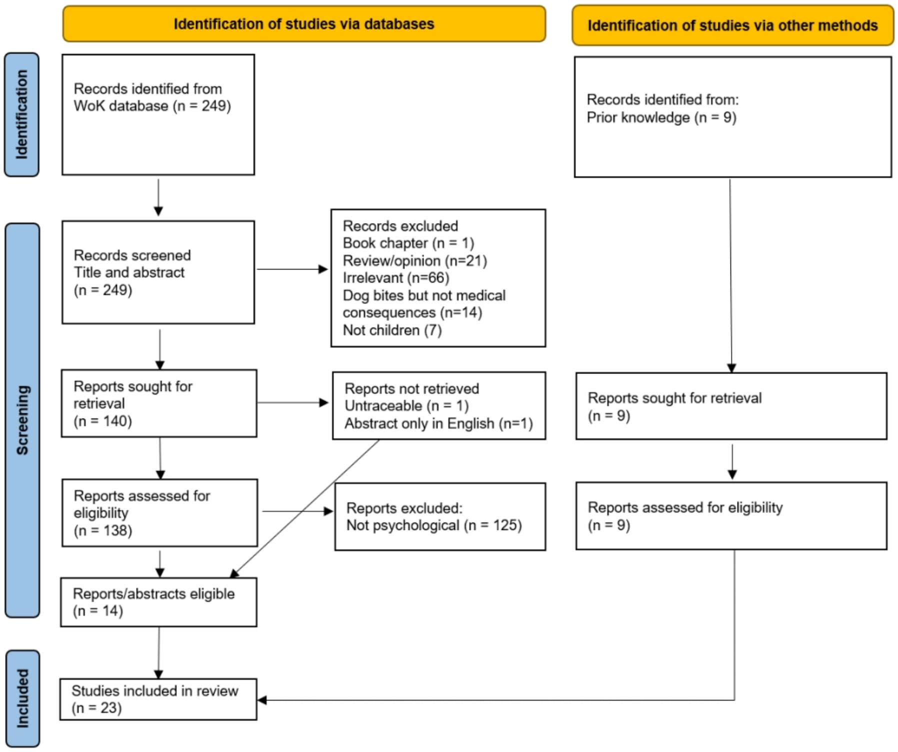

Figure 1. Flow chart of study search and screening process for studies of psychological consequences of dog bites to children

558x448mm (59 x 59 DPI)

BMJ Paediatrics Open

A review of psychological effects of dog bites in children

Journal:	*BMJ Paediatrics Open*
Manuscript ID	bmjpo-2020-000922.R1
Article Type:	Review
Date Submitted by the Author:	16-May-2024
Complete List of Authors:	Westgarth, Carri; University of Liverpool Faculty of Health and Life Sciences, Department of Livestock and One Health; University of Liverpool<br>Provazza, Serena; Alder Hey Children's NHS Foundation Trust, Department of Clinical Health Psychology<br>Nicholas, Jade; Independent Researcher<br>Gray, Victoria; Alder Hey Children's Hospital NHS Foundation Trust, Department of Clinical health Psychology
Keywords:	Child Health, Adolescent Health, Psychology

SCHOLARONE™
Manuscripts

I, the Submitting Author has the right to grant and does grant on behalf of all authors of the Work (as defined in the below author licence), an exclusive licence and/or a non-exclusive licence for contributions from authors who are: i) UK Crown employees; ii) where BMJ has agreed a CC-BY licence shall apply, and/or iii) in accordance with the terms applicable for US Federal Government officers or employees acting as part of their official duties; on a worldwide, perpetual, irrevocable, royalty-free basis to BMJ Publishing Group Ltd ("BMJ") its licensees and where the relevant Journal is co-owned by BMJ to the co-owners of the Journal, to publish the Work in this journal and any other BMJ products and to exploit all rights, as set out in our licence.

The Submitting Author accepts and understands that any supply made under these terms is made by BMJ to the Submitting Author unless you are acting as an employee on behalf of your employer or a postgraduate student of an affiliated institution which is paying any applicable article publishing charge ("APC") for Open Access articles. Where the Submitting Author wishes to make the Work available on an Open Access basis (and intends to pay the relevant APC), the terms of reuse of such Open Access shall be governed by a Creative Commons licence – details of these licences and which Creative Commons licence will apply to this Work are set out in our licence referred to above.

Other than as permitted in any relevant BMJ Author's Self Archiving Policies, I confirm this Work has not been accepted for publication elsewhere, is not being considered for publication elsewhere and does not duplicate material already published. I confirm all authors consent to publication of this Work and authorise the granting of this licence.

Confidential: For Review Only

A review of psychological effects of dog bites in children

Westgarth, Carri[1]., Provazza, Serena[2]., Nicholas, Jade.[3], Gray, Victoria[2].

1 Department of Livestock and One Health, Institute of Infection, Veterinary and Ecological Sciences, University of Liverpool, Leahurst Campus, Neston, Cheshire, CH66 1QU, UK

2 Department of Clinical Health Psychology, Alder Hey Children's NHS Foundation Trust, Eaton Road, Liverpool, Merseyside, L12 2AP, UK.

3 Independent Researcher, Hampshire, UK.

ABSTRACT

Background

Dog bites are a concerning health problem in children and one of the leading causes of non-fatal injuries in this population. Dog attacks not only cause physical injuries but can also lead to long-term psychological problems. A review was performed to investigate the scope of literature on psychological effects of dog bites on a paediatric population.

Methods

A literature search was performed on Web of Knowledge database between 1982-June 2023, returning 249 results. 14 primary studies reporting the psychological consequences of dog bites in children or adolescents were classed as eligible and 9 further studies added from prior knowledge and bibliographical searches. 23 studies involving 1894 participants met the criteria and were included in this review.

Results

Of these 23 studies, 8 were case studies or small case series reports (up to n=4), 14 larger descriptive studies, and 1 analytical cross-sectional study. There was a mixture of retrospective and prospective data-gathering. The most common psychological consequences of dog bites in children were post-traumatic stress disorder, dog phobia, nightmares and symptoms of anxiety and avoidance behaviours.

Confidential: For Review Only

Conclusions

Studies on dog bites in a paediatric population with a specific focus on the psychological consequences associated with dog bites and their management are sparse. Future research and practice should more greatly consider the psychological impact in child victims of dog bites and their family members, as well as their management to avoid development of mental health issues and improve their quality of life. Future research also needs to ascertain the efficacy of using Virtual Reality in treating children with dog phobia.

KEY MESSAGES

- Psychological consequences of dog bites to children are common but under-researched and often overlooked.
- Typical consequences of dog bites are post-traumatic stress disorder, dog phobia, nightmares and symptoms of anxiety and avoidance behaviours.
- Treatment of dog phobias can be challenging to deliver.
- Virtual reality offers a potential ethical and safe, controlled environment for cognitive behavioural therapy regarding dogs.

INTRODUCTION

Dog ownership is argued to provide some health benefits [1-3], however, dogs, as well as other animals, may cause severe injuries to humans. Although there are no global estimates of dog bites incidence, the World Health Organization reports dog bites account for tens of millions of injuries annually [4]. As a result, dog bites represent a major public health concern, affecting predominately the paediatric population [5]. In the UK, incidence of hospital admissions for dog bites in children (14 or under) is stable at approximately 14.4 per 100,000 population per year [5].

Confidential: For Review Only

It is well established that most of the child dog bite victims are bitten inside the home, and most dogs involved are known to the victim [6 7]. The COVID-19 global pandemic and the subsequent implementation of lockdowns with a 'stay at home' order forced children to spend more time than usual at home with a significant increase in dog bites [8 9].

Some evidence supports that children are more vulnerable to dog attacks compared to adults because of their smaller physical size, their underestimation of risk and their tendency to behave more impulsively [10], as well as leaning-in behaviour with animals in young children [11]. Rates of admission peak in the 5-9 age group [8]. Furthermore, children are often injured in the neck and head regions and many have facial wounds [12-14]. The younger the child the more frequent the bites are to the face [14 15] and young children have more extensive/severe injuries [13 16].

In addition to physical impact, dog bites often carry health care costs [8]. They also produce psychological costs to the victims and other parties involved [17-19], however, compared to physical, psychological consequences of dog bites and their management is still scarcely investigated and poorly reported [20 21] Timely recognition of psychological symptoms caused by dog attacks in children and subsequent psychological support to the child victims and their families would be of critical importance to prevent development of future mental health issues, which would in turn influence the quality of life of the children and their caretakers. The aim of this systematic review was to explore current evidence on the psychological impact of dog bites in children and adolescents.

METHODS

Protocol and inclusion criteria

This review was conducted according to the standards established by the Preferred Reporting Items for Systematic Reviews [22].

Confidential: For Review Only

A search of Web of Science Core Collection database was performed to identify original research articles focusing on psychological consequences of dog bite injuries in the paediatric population from 1982- June 2023. This single popular database was chosen due to time constraints and its broad coverage of science, social sciences and arts and humanities. The search term used on all fields was:

("child* OR pediatric") AND (dog* OR cani*) AND (bit* OR attack*) AND (psychological OR PTSD OR phobia OR trauma* OR anxiety)

At least the abstract needed to be available in English for assessment for relevant inclusion, and screening was performed by CW and SP. Articles were excluded if: they were not an original primary source of evidence in a scientific journal (ie book chapter or review papers were excluded); the study population did not include children (ie under 18 years of age, although it could also include adults); studies focused on bites in general (e.g., human bites, cat bites etc) rather than dog; the information reported was not specific to psychological effects of dog bites but rather on dog bites in general (e.g., wound management, dog bite incidence etc). Articles were excluded based on reading the full paper if the title or abstract did not already clearly exclude it; if the study purpose and population was deemed relevant, often the full paper needed to be read in order to find if psychological effects had been reported. Further relevant studies were also identified from bibliographic searches and prior knowledge. See Figure 1 for study search and inclusion flowchart. Relatively few studies met the inclusion criteria and thus sample size was low. In addition, the study designs found would be classed as low-level of evidence (such as case studies, descriptive case series and cross-sectional studies). For these reasons all studies and their findings have been reported here rather than exclusion based on quality. Evaluation and summary of the study design and psychological findings was performed by CW and JN.

https://mc.manuscriptcentral.com/bmjpo

Confidential: For Review Only

RESULTS

Out of 249 papers screened for eligibility, 14 studies met the criteria and were included in this review, along with 9 other studies identified through prior knowledge and bioblographies, totalling 23 studies involving 1894 participants (Figure 1). Of these 23 studies, eight were single case studies or small case series (up to 4 children per report) [23-30] (Supplementary Materials Table 1). Thirteen were descriptive studies of larger patient groups [7 15 18 19 31-39], one was a case-control study but the psychological findings were only descriptional [40], and one was an analytical cross-sectional study [41] (Supplementary Materials Table 2 and 3). A mixture of retrospective (ie incidental review of past case records, survey regarding fear of dogs, see Supplementary Materials Table 2) and prospective data collection (deliberate interview of dog bite cases as they attended hospital, follow-up interview of a cohort of bitten children, see Supplementary Materials Table 3) methods were reported depending on the study, and sometimes both.

Effects on children

One study specific to dog bite patients estimated that 70% of children had demonstrated concerning behaviours since the incident [37]. Post-traumatic Stress Disorder, (PTSD) was mentioned in eight studies: 2 of 4 children (a family in which one child was bitten) [23], diagnosed in a case study [28], diagnosed in descriptive studies of dog bite patients at a prevalence of 12/22 [31], 12/22 [19], 19/358 [18]; 98% within 5 days of a traumatic facial incident some of which were dog bites [35], and also mentioned more generally in two other studies, one which included (16%) child dog bite patients [34] and one specifically of child dog bites [36]. PTSD was typically diagnosed through use of a screening questionnaire or meeting six major criteria under DSM-IV. Acute Stress Disorder (ASD) was also mentioned (38/358 patients in [18]) and observed to be an early indicator of PTSD. In a study of children interviewed after a traumatic experience (16% of which were dog bites), PTSD symptoms were greater in the dog attack and parental violence groups than the 'mild stressor' group, and mothers of bitten children were also observed to develop PTSD which was associated with child

Confidential: For Review Only

PTSD score as reported by the parent [34]. In addition, in the dog attack group mothers observed girls to have greater PTSD symptoms and boys to have greater dissociative symptoms, while child self-report showed boys to have greater PTSD symptoms. Other studies did not find evidence of gender/age effects in regards to PTSD in child dog bites [18 31] The severity of the symptoms seemed to be related to the severity of the attacks; children who sustained more physically severe or multiple bites were more likely to develop PTSD symptoms [18 19 31].

Other than formally reporting PTSD/ASD, specific symptoms most commonly described in the articles were fearfulness/avoidance of dogs [24 25 27 30 33 36-39] and sleep disturbance/nightmares [23-25 31 36-38 40]. Other symptoms included bed wetting [23 25], selective mutism [28], traumatic memories/re-living [19 28 31], talking a lot about the incident [37], increased arousal [19 31], anger/aggression [24 31], withdrawn/depressed/numbing [19 28 31 35], fearful [37], panic attacks [27], anxiety [35 36], hypervigilance [31], difficulty playing/avoidance of playing outdoors [24 30], anxiety about hospitals [37], fear of dying or re-injury [35], and phobia of own image [36].

Effects on parents

Notably, parents reported changes in their behaviours as well, proving that dog bites represent a burden not only for the victims but also for their family [34 37] Parents described feeling shaken [25], guilty, fearful for child's safety and worried about scars [37].

Treatment of children

Despite the reporting of psychological symptoms after dog bites in children, it was rare for psychological treatment to be recorded; although it featured in case studies [23 25 27-30], when evaluating descriptive studies it was only reported in 2/100 [15], 2/277 [7], 2/38 [40] patients. Indeed, two studies specifically noted no children received any psychological services (0/22 [19]; 0/34 [37]) but 50% of parents felt it would be helpful [37].

Confidential: For Review Only

Other observations

Hon et al (2007) acknowledged that one challenge to collecting these data is that in an emergency department service where dog bites present is it might be difficult to gather information about psychological and emotional trauma [21]. Clearly there is an unidentified gap in both the literature and patient need for psychological assessment. In addition it has been suggested that interventions should also include educational programmes on the risk and severity of dog attacks offered to parents and children, given dog bites can have significant psychological consequences that can negatively impact on children quality of life [29].

DISCUSSION

The main purpose of this review was to explore the current literature on paediatric dog bites, with a specific focus on the psychological impacts of dog bites in children, as the psychological consequences of dog bites as well as their treatment are often overlooked [20 21]. Evidence suggested that children bitten by dogs are at high risk of developing psychological disturbances, ranging from fear of dogs to clinical PTSD, however, few studies mentioned psychological management or treatment offered after dog attacks. It appears that assessing children in an emergency department (ED) to evaluate psychological sequelae following dog bites, and further offering them psychological support, can be difficult and/or lacks consideration. This may result in psychological symptoms significantly impacting children and potentially worsening. A form of follow-up assessment is likely to be required to assess whether initial symptoms of psychological distress have settled, but whom this responsibility falls to once a patient is discharged from the ED, is unclear. Further, not only did dog injuries have an impact on the victims, but they also affected their carers. Psychological symptoms showed by young victims of dog bites seemed to be long lasting with some studies reporting over 12 months. Age and gender differences were inconclusive between

Confidential: For Review Only

studies but across studies more severe bites commonly resulted in more serious psychological impact.

It has become increasingly recognised that there is a need and opportunity to evaluate victims of traumatic injuries for psychological distress so that they can be appropriately referred on. For example this can occur during physical treatment such as plastic surgery.[42] However, t is clear from our review that many children who have been bitten by dogs or are otherwise fearful of dogs may benefit from psychological screening and if required, treatment, but this does not appear to be regularly occurring in practice. This is particularly concerning given children are in a crucial developmental stage with varied levels of supportive systems and resilience, and PTSD is a common psychiatric disorder after a child has a traumatic experience.[43]

Although different approaches have been found to be effective in treating results of trauma and in particular phobia of dogs in children, CBT (cognitive behavioural therapy) regarding some form of graded exposure appears to be the most widely utilised [44 45]. A difficulty with this is the ethical and practical safety implications regarding controlled exposure to real animals who can be difficult to access, unpredictable and to some extent uncontrollable in their behaviours, and with their own welfare considerations [46]. For example, one intervention states "*Parents were not provided with explicit instructions on how to find dogs but were encouraged to consider various options: dogs owned by extended family members, neighbors, and friends; dogs at pet stores, breeders, and animal shelters; dogs at the park or other recreation areas*" [45]. Another play-based intervention used dogs with handlers within the intensive session, with no description about how these had been assessed for suitability, and "*during the session, the child was encouraged to engage in a range of different tasks with each dog including approaching and patting the dog, offering the dog treats and walking the dog on the lead*" [44]. Great care must be taken in order to be able to gradually control the intensity of exposure and also safeguard both child and animal welfare during such treatment so that

Confidential: For Review Only

neither becomes overwhelmed and both have a positive experience. This includes providing very clear guidance to those delivering it, including parents.

A potential more ethical solution to these challenges during the intensive and early stages of therapy would be to use virtual reality [46]. This would allow a gradual and controlled increase in exposure to particular dog behaviours required for that case. For example, Farrel (2021) have suggested the use of Virtual Reality therapy in treating dog phobia can be possible using just one treatment session [47]. In eight children with specific phobia of dogs, after one month follow-up, 75% of the children were considered recovered. The authors concluded that VR can be effectively be used as an alternative to the classic in-vivo exposure-based therapy and might overcome some of its challenges as the most difficult CBT technique to deliver. More research is needed to confirm the efficacy of VR as an effective treatment of dog fear in a paediatric population.

This review has the merit of investigating and summarising existing evidence on psychological effects of paediatric dog bites and their management, as to the best of our knowledge, such investigation has not been conducted before. However, it presents with some limitations. First of all, even though the literature search was performed on a highly relevant database, it was only a single database and some studies may be missing from this review. Additionally, of the few studies identified, many had a very limited sample size, which may limited the statistical power and generalizability of the findings. They also mostly used study designs with a high risk of bias, such as case studies, and descriptive cross-sectional studies, often with retrospective data collection simply reviewing case notes. For example, unless it was particularly noted because a parent or child raised the issue, psychological impacts may have remained unreported. Those studies of a potentially lower risk of bias that used prospective data collection still varied in quality of screening methods for psychological outcomes and whether follow-up was used. Therefore, the conclusions

Confidential: For Review Only

drawn here should be taken with caution. Notwithstanding the above limitations, this review provides an insight into the psychological consequences of paediatric dog bites.

To conclude, dog bites in children represent a traumatic event, which can cause devasting psychological consequences in the victims and in their families. A thorough investigation of the psychological impact of dog bites on children and their parents followed by a prompt multidisciplinary managenent of both physical and psychological symptoms would lead to better outcomes, preventing the occurrence of more severe mental health problems such as phobia and PTSD and improve the quality of life of the victims and their families. Furthermore, educational programmes on the risk and severity of dog attacks should be offered to parents and children, to prevent dog bites. Cognitive behavioural therapy can be used to treat fear of dogs in children, with VR representing an alternative to the classic in vivo exposition therapy that requires more investigation. Future research should focus more on the psychological impact of dog injuries and on the treatment of child victims of dog bites and their family members, to avoid development of mental health issues and improve their quality of life.

FUNDING

This work was supported by the Faculty of Health and Life Sciences, University of Liverpool.

COMPETING INTERESTS

CW is a member of the Merseyside Dog Safety Partnership, which has a website containing useful resources for dog bite prevention. www.merseydogsafe.co.uk. There are no other competing interests to declare.

RESEARCH ETHICS APPROVAL

Not applicable - this study does not involve human participants.

CONTRIBUTORS

Confidential: For Review Only

CW conceptualised the paper. CW and SP performed the searches and paper selection. CW and JN reviewed the papers and assimilated the data. CW and SP wrote the first draft. All authors including VG commented on and revised the paper. CW submitted the paper. CW is responsible for the content as guarantor.

DATA AVAILABILITY

No other datasets were generated.

PATIENT CONSENT FOR PUBLICATION

Not applicable.

PROVENANCE AND PEER REVIEW

Commissioned; externally peer reviewed.

REFERENCES

1. Herzog H. The Impact of Pets on Human Health and Psychological Well-Being: Facto, Fiction, or Hypothesis? *Current Directions in Psychological Science* 2011;20:236-39.
2. O'Haire M. Companion animals and human health: Benefits, challenges, and the road ahead. *Journal of Veterinary Behaviour* 2010;5:226-34.
3. Rodriguez KE, Herzog H, Gee NR. Variability in Human-Animal Interaction Research. *Front Vet Sci* 2021;7:9. doi: 10.3389/fvets.2020.619600
4. WHO. Animal Bites: World Health Organisation; 2018 [cited 2024 9th January]. Available from: https://www.who.int/news-room/fact-sheets/detail/animal-bites.
5. Tulloch JSP, Owczarczak-Garstecka SC, Fleming KM, et al. English hospital episode data analysis (1998–2018) reveal that the rise in dog bite hospital admissions is driven by adult cases. *Scientific Reports* 2021;11(1):1767. doi: 10.1038/s41598-021-81527-7
6. Cornelissen JMR, Hopster H. Dog bites in The Netherlands: A study of victims, injuries, circumstances and aggressors to support evaluation of breed specific legislation. *Veterinary Journal* 2010;186(3):292-98. doi: 10.1016/j.tvjl.2009.10.001
7. Chiam SC, Solanki NS, Lodge M, et al. Retrospective review of dog bite injuries in children presenting to a South Australian tertiary children's hospital emergency department. *J Paediatr Child Health* 2014;50(10):791-4. doi: 10.1111/jpc.12642 [published Online First: 2014/07/22]
8. Tulloch JSP, Minford S, Pimblett V, et al. Paediatric emergency department dog bite attendance during the COVID-19 pandemic: an audit at a tertiary children's hospital. *BMJ paediatrics open* 2021;5(1):e001040-e40. doi: 10.1136/bmjpo-2021-001040
9. Parente G, Gargano T, Di Mitri M, et al. Consequences of COVID-19 Lockdown on Children and Their Pets: Dangerous Increase of Dog Bites among the Paediatric Population. *Children (Basel)* 2021;8(8) doi: 10.3390/children8080620 [published Online First: 2021/08/28]
10. Shen J, Rouse J, Godbole M, et al. Systematic Review: Interventions to Educate Children About Dog Safety and Prevent Pediatric Dog-Bite Injuries: A Meta-Analytic Review. *J Pediatr Psychol* 2017;42(7):779-91. doi: 10.1093/jpepsy/jsv164

Confidential: For Review Only

11. Meints K, Syrnyk C, De Keuster T. Why do children get bitten in the face? *Injury Prevention* 2010;16(Suppl 1):A172-A73. doi: 10.1136/ip.2010.029215.617
12. Kasbekar AV, Garfit H, Duncan C, et al. Dog bites to the head and neck in children; an increasing problem in the UK. *Clinical Otolaryngology* 2013;38(3):259-62. doi: 10.1111/coa.12094
13. Khan K, Horswell BB, Samanta D. Dog-Bite Injuries to the Craniofacial Region: An Epidemiologic and Pattern-of-Injury Review at a Level 1 Trauma Center. *Journal of Oral and Maxillofacial Surgery* 2020;78(3):401-13. doi: https://doi.org/10.1016/j.joms.2019.11.002
14. Sribnick EA, Sarda S, Sawvel MS, et al. Dog bite injuries in children: Clinical implications for head involvement. *Trauma* 2016;18(3):210-15. doi: 10.1177/1460408616631772
15. Kahn A, Bauche P, Lamoureux J, et al. Child victims of dog bites treated in emergency departments: a prospective survey. *European Journal of Pediatrics* 2003;162(4):254-58. doi: 10.1007/s00431-002-1130-6
16. Brogan TV, Bratton SL, Dowd MD, et al. SEVERE DOG BITES IN CHILDREN. *Pediatrics* 1995;96(5):947-50.
17. Westgarth C, Watkins F. Chapter 23: Impact of dog aggression on victims. In: Mills DS, Westgarth C, eds. Dog Bites: A Multidisciplinary Perspective. 1st Edition ed. Sheffield: 5M Publishing 2017:309-20.
18. Ji L, Xiaowei Z, Chuanlin W, et al. Investigation of posttraumatic stress disorder in children after animal-induced injury in China. *Pediatrics* 2010;126(2):e320-4. doi: 10.1542/peds.2009-3530 [published Online First: 2010/07/14]
19. Peters V, Sottiaux M, Appelboom J, et al. Posttraumatic stress disorder after dog bites in children. *The Journal of Pediatrics* 2004;144(1):121-22. doi: http://dx.doi.org/10.1016/j.jpeds.2003.10.024
20. Jakeman M, Oxley JA, Owczarczak-Garstecka SC, et al. Pet dog bites in children: management and prevention. *BMJ Paediatrics Open* 2020;4(1):e000726. doi: 10.1136/bmjpo-2020-000726
21. Hon KLE, Fu CCA, Chor CM, et al. Issues associated with dog bite injuries in children and adolescents assessed at the emergency department. *Pediatric Emergency Care* 2007;23(7):445-49.
22. Page MJ, McKenzie JE, Bossuyt PM, et al. The PRISMA 2020 statement: an updated guideline for reporting systematic reviews. *BMJ* 2021;372:n71. doi: 10.1136/bmj.n71
23. Albano AM, Miller PP, Zarate R, et al. Behavioral assessment and treatment of PTSD in prepubertal children: Attention to developmental factors and innovative strategies in the case study of a family. *Cognitive and Behavioral Practice* 1997;4(2):245-62. doi: https://doi.org/10.1016/S1077-7229(97)80003-X
24. Drell MJ, Gaensbauer TJ, Siegel CH, et al. Clinical round table: A case of trauma to a 21-month-old girl. *Infant Mental Health Journal* 1995;16(4):318-33. doi: https://doi.org/10.1002/1097-0355(199524)16:4<318::AID-IMHJ2280160407>3.0.CO;2-L
25. Gislason IL, Call JD. Dog Bite in Infancy: Trauma and Personality Development. *Journal of the American Academy of Child Psychiatry* 1982;21(2):203-07. doi: https://doi.org/10.1016/S0002-7138(09)60921-3
26. Macias D, Kwon DI, Walker PC, et al. Microvascular replantation of a composite facial avulsion in a 24-month-old child after dog bite. *Microsurgery* 2018;38(2):218-21. doi: https://doi.org/10.1002/micr.30275
27. Saha S. Life-Threatening Panfacial Wild Dog Bites in a Child. *Wilderness & Environmental Medicine* 2021;32(4):511-16. doi: https://doi.org/10.1016/j.wem.2021.07.001
28. Anyfantakis D, Botzakis E, Mplevrakis E, et al. Selective mutism due to a dog bite trauma in a 4-year-old girl: A case report. *Journal of Medical Case Reports* 2009;3

29. de Carvalho MF, Hardtke LA, de Souza MF, et al. Comminuted mandibular fracture in child victim of dog bite. *Dent Traumatol* 2012;28(4):324-8. doi: 10.1111/j.1600-9657.2011.01079.x [published Online First: 2011/11/22]
30. Glasscock SE, MacLean Jr WE. Use of Contact Desensitization and Shaping in the Treatment of Dog Phobia and Generalized Fear of the Outdoors. *Journal of Clinical Child Psychology* 1990;19(2):169-71. doi: 10.1207/s15374424jccp1902_9
31. De Keuster T, Lamoureux J, Kahn A. Epidemiology of dog bites: A Belgian experience of canine behaviour and public health concerns. *Veterinary Journal* 2006;172(3):482-87.
32. Hersant B, Cassier S, Constantinescu G, et al. Facial dog bite injuries in children: Retrospective study of 77 cases. *Annales De Chirurgie Plastique Esthetique* 2012;57(3):230-39. doi: 10.1016/j.anplas.2011.11.003
33. Pédrono G, Ricard C, Bouilly M, et al. Assessment of 16-month Sequelae Due to Dog Bites Originally Studied in a French Multicenter Survey from 2009 to 2011. *Wounds* 2018;30(5):84-89. [published Online First: 2018/03/10]
34. Rossman BR, Bingham RD, Emde RN. Symptomatology and adaptive functioning for children exposed to normative stressors, dog attack, and parental violence. *J Am Acad Child Adolesc Psychiatry* 1997;36(8):1089-97. doi: 10.1097/00004583-199708000-00016 [published Online First: 1997/08/01]
35. Rusch MD, Grunert BK, Sanger JR, et al. Psychological adjustment in children after traumatic disfiguring injuries: A 12-month follow-up. *Plastic and Reconstructive Surgery* 2000;106(7):1451-58.
36. Touré G, Angoulangouli G, Méningaud J-P. Epidemiology and classification of dog bite injuries to the face: A prospective study of 108 patients. *Journal of Plastic, Reconstructive & Aesthetic Surgery* 2015;68(5):654-58. doi: https://doi.org/10.1016/j.bjps.2015.01.001
37. Boat BW, Dixon CA, Pearl E, et al. Pediatric dog bite victims: a need for a continuum of care. *Clin Pediatr (Phila)* 2012;51(5):473-7. doi: 10.1177/0009922811435504 [published Online First: 2012/02/02]
38. Schalamon J, Ainoedhofer H, Singer G, et al. Analysis of dog bites in children who are younger than 17 years. *Pediatrics* 2006;117(3):E374-E79. doi: 10.1542/peds.2005-1451
39. Shen J, Li S, Xiang H, et al. Antecedents and consequences of pediatric dog-bite injuries and their developmental trends: 101 cases in rural China. *Accident Analysis and Prevention* 2014;63:22-29.
40. Parent B, Bykowski MR, Marji FP, et al. Pediatric Craniofacial Fractures From Canine Bites. *J Craniofac Surg* 2021;32(4):1627-32. doi: 10.1097/scs.0000000000007546 [published Online First: 2021/03/21]
41. Doogan S, Thomas GV. Origins of fear of dogs in adults and children: The role of conditioning processes and prior familiarity with dogs. *Behaviour Research and Therapy* 1992;30(4):387-94. doi: https://doi.org/10.1016/0005-7967(92)90050-Q
42. Rusch MD, Gould LJ, Dzwierzynski WW, et al. Psychological Impact of Traumatic Injuries: What the Surgeon Can Do. *Plastic and Reconstructive Surgery* 2002;109(1):18-24.
43. Caffo E, Belaise C. Psychological aspects of traumatic injury in children and adolescents. *Child Adolesc Psychiatr Clin N Am* 2003;12(3):493-535. doi: 10.1016/s1056-4993(03)00004-x [published Online First: 2003/08/13]
44. Farrell LJ, Kershaw H, Ollendick T. Play-Modified One-Session Treatment for Young Children with a Specific Phobia of Dogs: A Multiple Baseline Case Series. *Child Psychiatry Hum Dev* 2018;49(2):317-29. doi: 10.1007/s10578-017-0752-x [published Online First: 2017/08/03]
45. Radtke SR, Muskett A, Coffman MF, et al. Bibliotherapy for Specific Phobias of Dogs in Young Children: A Pilot Study. *Journal of Child and Family Studies* 2023;32(2):373-83. doi: 10.1007/s10826-022-02304-2

46. Oxley JA, Santa K, Meyer G, et al. A Systematic Scoping Review of Human-Dog Interactions in Virtual and Augmented Reality: The Use of Virtual Dog Models and Immersive Equipment. *Frontiers in Virtual Reality* 2022;3 doi: 10.3389/frvir.2022.782023
47. Farrell LJ, Miyamoto T, Donovan CL, et al. Virtual Reality One-Session Treatment of Child-Specific Phobia of Dogs: A Controlled, Multiple Baseline Case Series. *Behavior Therapy* 2021;52(2):478-91. doi: https://doi.org/10.1016/j.beth.2020.06.003

Confidential: For Review Only

FIGURES

Figure 1. Flow chart of study search and screening process for studies of psychological consequences of dog bites to children

Confidential: For Review Only

TABLES

Confidential: For Review Only

A review of p~~P~~sychological effects of dog bites in children

Westgarth, Carri[1]., Provazza, Serena[2]., Nicholas, Jade.[3], Gray, Victoria[2].

1 Department of Livestock and One Health, Institute of Infection, Veterinary and Ecological Sciences, University of Liverpool, Leahurst Campus, Neston, Cheshire, CH66 1QU, UK

2 Department of Clinical Health Psychology, Alder Hey Children's NHS Foundation Trust, Eaton Road, Liverpool, Merseyside, L12 2AP, UK.

3 Independent Researcher, Hampshire, UK.

Confidential: For Review Only

ABSTRACT

Background

Dog bites are a concerning health problem in children and one of the leading causes of non-fatal injuries in this population. Dog attacks not only cause physical injuries but can also lead to long-term psychological problems. A ~~systematic~~ review was performed to investigate the scope of literature on psychological effects of dog bites on a paediatric population.

Methods

A literature search was performed on Web of Knowledge database between 1982-June 2023, returning 249 results. 14 primary studies reporting the psychological consequences of dog bites in children or adolescents were classed as eligible and 9 further studies added from prior knowledge and bibliographical searches. 23 studies involving 1894 participants met the criteria and were included in this review.

Results

Of these 23 studies, 8 were case studies or small case series reports (up to n=4), 14 larger descriptive studies, and 1 analytical cross-sectional study. There was a mixture of retrospective and prospective data-gathering. The most common psychological consequences of dog bites in children were post-traumatic stress disorder, dog phobia, nightmares and symptoms of anxiety and avoidance behaviours.

Conclusions

Studies on dog bites in a paediatric population with a specific focus on the psychological ~~consequeces~~consequences associated with dog bites and their management are sparse. Future research and practice should more greatly consider the psychological impact in child victims of dog bites and their family members, as well as their management to avoid development of mental health issues and improve their quality of life. Future research also needs to ascertain the efficacy of using Virtual Reality in treating children with dog phobia.

KEY MESSAGES

- Psychological consequences of dog bites to children are common but under-researched and often overlooked.
- Typical consequences of dog bites are post-traumatic stress disorder, dog phobia, nightmares and symptoms of anxiety and avoidance behaviours.
- Treatment of dog phobias can be challenging to deliver.
- Virtual reality offers a potential ethical and safe, controlled environment for cognitive behavioural therapy regarding dogs.

INTRODUCTION

Dog ownership is argued to provide some health benefits [1-3], however, dogs, as well as other animals, may cause severe injuries to humans. Although there are no global estimates of dog bites incidence, the World Health Organization reports dog bites account for tens of millions of injuries annually [4]. As a result, dog bites represent a major public health concern, affecting predominately the paediatric population [5]. In the UK, incidence of hospital admissions for dog bites in children (14 or under) is stable at approximately 14.4 per 100,000 population per year [5].

Confidential: For Review Only

It is well established that most of the child dog bite victims are bitten inside the home, and most dogs involved are known to the victim [6 7]. The COVID-19 global pandemic and the subsequent implementation of lockdowns with a 'stay at home' order forced children to spend more time than usual at home with a significant increase in dog bites [8 9].

Some evidence supports that children are more vulnerable to dog attacks compared to adults because of their smaller physical size, their underestimation of risk and their tendency to behave more impulsively [10], as well as leaning-in behaviour with animals in young children [11]. Rates of admission peak in the 5-9 age group [8]. Furthermore, children are often injured in the neck and head regions and many have facial wounds [12-14]. The younger the child the more frequent the bites are to the face [14 15] and young children have more extensive/severe injuries [13 16].

In addition to physical impact, dog bites often carry health care costs [8]. They also produce psychological costs to the victims and other parties involved [17-19], however, compared to physical, psychological consequences of dog bites and their management is still scarcely investigated and poorly reported [20 21] Timely recognition of psychological symptoms caused by dog attacks in children and subsequent psychological support to the child victims and their families would be of critical importance to prevent development of future mental health issues, which would in turn influence the quality of life of the children and their caretakers. The aim of this systematic review was to explore current evidence on the psychological impact of dog bites in children and adolescents.

METHODS

Protocol and inclusion criteria

This ~~systematic~~ review was conducted according to the standards established by the Preferred Reporting Items for Systematic Reviews [22].

Confidential: For Review Only

A search of ~~Web of Knowledge~~<u>Web of Science Core Collection</u> database was performed to identify original research articles focusing on psychological consequences of dog bite injuries in the paediatric population from 1982- June 2023. <u>This single popular database was chosen due to time constraints and its broad coverage of science, social sciences and arts and humanities.</u> The search term used on all fields was:

("child* OR pediatric") AND (dog* OR cani*) AND (bit* OR attack*) AND (psychological OR PTSD OR phobia OR trauma* OR anxiety)

At least the abstract needed to be available in English for assessment for relevant inclusion, and screening was performed by CW and SP. Articles were excluded if: they were not an original primary source of evidence in a scientific journal (ie book chapter or review papers were excluded); the study population did not include children (ie under 18 years of age, although it could also include adults); studies focused on bites in general (e.g., human bites, cat bites etc) rather than dog; the information reported was not specific to psychological effects of dog bites but rather on dog bites in general (e.g., wound management, dog bite incidence etc). Articles were excluded based on reading the full paper if the title or abstract did not already clearly exclude it; if the study purpose and population was deemed relevant, often the full paper needed to be read in order to find if psychological effects had been reported. Further relevant studies were also identified from bibliographic searches and prior knowledge. See Figure 1 for study search and inclusion flowchart. ~~Due to the low number of~~<u>Relatively few</u> studies ~~that~~ met the inclusion criteria <u>and thus sample size was low. In addition, the study designs found would be classed as</u> ~~and~~ low-level of evidence ~~study designs used (~~<u>(such as</u> case studies ~~and~~<u>,</u> descriptive case series <u>and cross-sectional studies</u>)<u>. For these reasons</u>~~,~~ all studies <u>and their findings</u> have been reported here rather than exclusion based on quality. Evaluation and summary of the study design and psychological findings was performed by CW and JN.

Confidential: For Review Only

RESULTS

Out of 249 papers screened for eligibility, 14 studies met the criteria and were included in this review, along with 9 other studies identified through prior knowledge and bioblographies, totalling 23 studies involving 1894 participants (Figure 1). Of these 23 studies, eight were single case studies or small case series (up to 4 children per report~~-~~) [23-30] (Supplementary Materials Table 1). Thirteen were descriptive studies of larger patient groups [7 15 18 19 31-39], one was a case-control study but the psychological findings were only descriptional [40], and one was an analytical cross-sectional study [41] (~~Table 2~~Supplementary Materials Table 2 and 3). A mixture of retrospective (ie incidental review of past case records, survey regarding fear of dogs, see Supplementary Materials Table 2) and prospective data collection (deliberate interview of dog bite cases as they attended hospital, follow-up interview of a cohort of bitten children, see Supplementary Materials Table 3) methods were reported depending on the study, and sometimes both.

~~Summary of the findings~~Effects on children

One study specific to dog bite patients estimated that 70% of children had demonstrated concerning behaviours since the incident [37]. Post-traumatic Stress Disorder ~~was commonly encountered~~, ~~being~~ (PTSD) was mentioned in eight studies: 2 of 4 children (a family in which one child was bitten) [23], diagnosed in a case study [28], diagnosed in descriptive studies of dog bite patients at a prevalence of 12/22 [31], 12/22 [19], 19/358 [18]; 98% within 5 days of a traumatic facial incident some of which were dog bites [35], and also mentioned more generally in two other studies, one which included (16%) child dog bite patients [34] and one specifically of child dog bites [36]. PTSD was typically diagnosed through use of a screening questionnaire or meeting six major criteria under DSM-IV. Acute Stress Disorder (ASD) was also mentioned (38/358 patients in [18]) and observed to be an early indicator of PTSD. In a study of children interviewed after a traumatic experience (16% of which were dog bites), PTSD symptoms were greater in the dog attack and parental violence groups than the 'mild

Confidential: For Review Only

stressor' group, and mothers of bitten children were also observed to develop PTSD which was associated with child PTSD score as reported by the parent [34]. In addition, in the dog attack group mothers observed girls to have greater PTSD symptoms and boys to have greater dissociative symptoms, while child self-report showed boys to have greater PTSD symptoms. Other studies did not find evidence of gender/age effects in regards to PTSD in child dog bites [18 31] The severity of the symptoms seemed to be related to the severity of the attacks; children who sustained more physically severe or multiple bites were more likely to develop PTSD symptoms [18 19 31].

Other than formally reporting PTSD/ASD, specific symptoms most commonly described in the articles were fearfulness/avoidance of dogs [24 25 27 30 33 36-39] and sleep disturbance/nightmares [23-25 31 36-38 40]. Other symptoms included bed wetting [23 25], selective mutism [28], traumatic memories/re-living [19 28 31], talking a lot about the incident [37], increased arousal [19 31], anger/aggression [24 31], withdrawn/depressed/numbing [19 28 31 35], fearful [37], panic attacks [27], anxiety [35 36], hypervigilance [31], difficulty playing/avoidance of playing outdoors [24 30], anxiety about hospitals [37], fear of dying or re-injury [35], and phobia of own image [36].

Effects on parents

Notably, parents reported changes in their behaviours as well, proving that dog bites represent a burden not only for the victims but also for their family [34 37] Parents described feeling shaken [25], guilty, fearful for child's safety and worried about scars [37].

Treatment of children

Despite the reporting of psychological symptoms after dog bites in children, it was rare for psychological treatment to be recorded; although it featured in case studies [23 25 27-30], when evaluating descriptive studies it was only reported in 2/100 [15], 2/277 [7], 2/38 [40] patients. Indeed, two

Confidential: For Review Only

studies specifically noted no children received any psychological services (0/22 [19]; 0/34 [37]) but 50% of parents felt it would be helpful [37].

Other observations

Hon et al (2007) acknowledged that one challenge to collecting these data is that in an emergency department service where dog bites present is it might be difficult to gather information about psychological and emotional trauma [21]. Clearly there is an unidentified gap in both the literature and patient need for psychological assessment. In addition it has been suggested that interventions should also include educational programmes on the risk and severity of dog attacks offered to parents and children, given dog bites can have significant psychological consequences that can negatively impact on children quality of life [29].

DISCUSSION

The main purpose of this review was to explore the current literature on paediatric dog bites, with a specific focus on the psychological impacts of dog bites in children, as the psychological consequences of dog bites as well as their treatment are often overlooked [20 21]. Evidence suggested that children bitten by dogs are at high risk of developing psychological disturbances, ranging from fear of dogs to clinical PTSD, however, few studies mentioned psychological management or treatment offered after dog attacks. It appears that assessing children in an emergency department (ED) to evaluate psychological sequelae following dog bites, and further offering them psychological support, can be difficult and/or lacks consideration. This may result in psychological symptoms significantly impacting children and potentially worsening. A form of follow-up assessment is likely to be required to assess whether initial symptoms of psychological distress have settled, but whom this responsibility falls to once a patient is discharged from the ED, is unclear. Further, not only did dog injuries have an impact on the victims, but they also affected their carers. Psychological symptoms showed by young victims of dog bites seemed to be long lasting with

Confidential: For Review Only

some studies reporting over 12 months. Age and gender differences were inconclusive between studies but across studies more severe bites commonly resulted in more serious psychological impact.

It has become increasingly recognised that there is a need and opportunity to evaluate victims of traumatic injuries for psychological distress so that they can be appropriately referred on. For example this can occur during physical treatment such as plastic surgery.[42] However, ~~I~~t is clear from our review that many children who have been bitten by dogs or are otherwise fearful of dogs may benefit from psychological screening and if required, treatment, but this does not appear to be regularly occurring in practice. ~~.~~This is particularly concerning given children are in a crucial developmental stage with varied levels of supportive systems and resilience, and PTSD is a common psychiatric disorder after a child has a traumatic experience.[43]

Although different approaches have been found to be effective in treating results of trauma and in particular phobia ~~for~~ of dogs in children, CBT (cognitive behavioural therapy) regarding some form of graded exposure appears to be the most widely utilised [44 45]. A difficulty with this is the ethical and practical safety implications regarding controlled exposure to real animals who can be difficult to access, unpredictable and to some extent uncontrollable in their behaviours, and with their own welfare considerations [46]. For example, one intervention states "*Parents were not provided with explicit instructions on how to find dogs but were encouraged to consider various options: dogs owned by extended family members, neighbors, and friends; dogs at pet stores, breeders, and animal shelters; dogs at the park or other recreation areas*" [45]. Another play-based intervention used dogs with handlers within the intensive session, with no description about how these had been assessed for suitability, and "*during the session, the child was encouraged to engage in a range of different tasks with each dog including approaching and patting the dog, offering the dog treats and walking the dog on the lead*" [44]. Great care must be taken in order to be able to gradually control the

Confidential: For Review Only

intensity of exposure and also safeguard both child and animal welfare during such treatment so that neither becomes overwhelmed and both have a positive experience. This includes providing very clear guidance to those delivering it, including parents.

A potential more ethical solution to these challenges during the intensive and early stages of therapy would be to use virtual reality [46]. This would allow a gradual and controlled increase in exposure to particular dog behaviours required for that case. For example, Farrel (2021) have suggested the use of Virtual Reality therapy in treating dog phobia can be possible using just one treatment session [47]. In eight children with specific phobia of dogs, after one month follow-up, 75% of the children were considered recovered. The authors concluded that VR can be effectively be used as an alternative to the classic in-vivo exposure-based therapy and might overcome some of its challenges as the most difficult CBT technique to deliver. More research is needed to confirm the efficacy of VR as an effective treatment of dog fear in a paediatric population.

This review has the merit of investigating and summarising existing evidence on psychological effects of paediatric dog bites and their management, as to the best of our knowledge, such investigation has not been conducted before. However, it presents with some limitations. First of all, even though the literature search was performed on a highly relevant database, it was only a single database and some studies may be missing from this review. Additionally, of the few studies identified, many had a very limited sample size, which may limited the statistical power and generalizability of the findings. They also mostly used study designs with a high risk of bias, such as case studies, and descriptive cross-sectional studies, often with retrospective data collection simply reviewing case notes. For example, unless it was particularly noted because a parent or child raised the issue, psychological impacts may have remained unreported. Those studies of a potentially lower risk of bias that used prospective data collection still varied in quality of screening methods for psychological outcomes and whether follow-up was used. Therefore, the conclusions

Confidential: For Review Only

drawn here should be taken with caution. Notwithstanding the above limitations, this review provides an insight into the psychological consequences of paediatric dog bites.

To conclude, dog bites in children represent a traumatic event, which can cause devasting psychological consequences in the victims and in their families. A thorough investigation of the psychological impact of dog bites on children and their parents followed by a prompt multidisciplinary managenent of both physical and psychological symptoms would lead to better outcomes, preventing the occurrence of more severe mental health problems such as phobia and PTSD and improve the quality of life of the victims and their families. Furthermore, educational programmes on the risk and severity of dog attacks should be offered to parents and children, to prevent dog bites. Cognitive behavioural therapy can be used to treat fear of dogs in children, with VR representing an alternative to the classic in vivo exposition therapy that requires more investigation. Future research should focus more on the psychological impact of dog injuries and on the treatment of child victims of dog bites and their family members, to avoid development of mental health issues and improve their quality of life.

Confidential: For Review Only

FUNDING

This work was supported by the Faculty of Health and Life Sciences, University of Liverpool.

COMPETING INTERESTS

CW is a member of the Merseyside Dog Safety Partnership, which has a website containing useful resources for dog bite prevention. www.merseydogsafe.co.uk. There are no other competing interests to declare.

RESEARCH ETHICS APPROVAL

Not applicable - this study does not involve human participants.

CONTRIBUTORS

CW conceptualised the paper. CW and SP performed the searches and paper selection. CW and JN reviewed the papers and assimilated the data. CW and SP wrote the first draft. All authors including VG commented on and revised the paper. CW submitted the paper. CW is responsible for the content as guarantor.

DATA AVAILABILITY

No other datasets were generated.

PATIENT CONSENT FOR PUBLICATION

Not applicable.

PROVENANCE AND PEER REVIEW

Commissioned; externally peer reviewed.

Confidential: For Review Only

REFERENCES

1. Herzog H. The Impact of Pets on Human Health and Psychological Well-Being: Facto, Fiction, or Hypothesis? *Current Directions in Psychological Science* 2011;20:236-39.
2. O'Haire M. Companion animals and human health: Benefits, challenges, and the road ahead. *Journal of Veterinary Behaviour* 2010;5:226-34.
3. Rodriguez KE, Herzog H, Gee NR. Variability in Human-Animal Interaction Research. *Front Vet Sci* 2021;7:9. doi: 10.3389/fvets.2020.619600
4. WHO. Animal Bites: World Health Organisation; 2018 [cited 2024 9th January]. Available from: https://www.who.int/news-room/fact-sheets/detail/animal-bites.
5. Tulloch JSP, Owczarczak-Garstecka SC, Fleming KM, et al. English hospital episode data analysis (1998–2018) reveal that the rise in dog bite hospital admissions is driven by adult cases. *Scientific Reports* 2021;11(1):1767. doi: 10.1038/s41598-021-81527-7
6. Cornelissen JMR, Hopster H. Dog bites in The Netherlands: A study of victims, injuries, circumstances and aggressors to support evaluation of breed specific legislation. *Veterinary Journal* 2010;186(3):292-98. doi: 10.1016/j.tvjl.2009.10.001
7. Chiam SC, Solanki NS, Lodge M, et al. Retrospective review of dog bite injuries in children presenting to a South Australian tertiary children's hospital emergency department. *J Paediatr Child Health* 2014;50(10):791-4. doi: 10.1111/jpc.12642 [published Online First: 2014/07/22]
8. Tulloch JSP, Minford S, Pimblett V, et al. Paediatric emergency department dog bite attendance during the COVID-19 pandemic: an audit at a tertiary children's hospital. *BMJ paediatrics open* 2021;5(1):e001040-e40. doi: 10.1136/bmjpo-2021-001040
9. Parente G, Gargano T, Di Mitri M, et al. Consequences of COVID-19 Lockdown on Children and Their Pets: Dangerous Increase of Dog Bites among the Paediatric Population. *Children (Basel)* 2021;8(8) doi: 10.3390/children8080620 [published Online First: 2021/08/28]
10. Shen J, Rouse J, Godbole M, et al. Systematic Review: Interventions to Educate Children About Dog Safety and Prevent Pediatric Dog-Bite Injuries: A Meta-Analytic Review. *J Pediatr Psychol* 2017;42(7):779-91. doi: 10.1093/jpepsy/jsv164

11. Meints K, Syrnyk C, De Keuster T. Why do children get bitten in the face? *Injury Prevention* 2010;16(Suppl 1):A172-A73. doi: 10.1136/ip.2010.029215.617
12. Kasbekar AV, Garfit H, Duncan C, et al. Dog bites to the head and neck in children; an increasing problem in the UK. *Clinical Otolaryngology* 2013;38(3):259-62. doi: 10.1111/coa.12094
13. Khan K, Horswell BB, Samanta D. Dog-Bite Injuries to the Craniofacial Region: An Epidemiologic and Pattern-of-Injury Review at a Level 1 Trauma Center. *Journal of Oral and Maxillofacial Surgery* 2020;78(3):401-13. doi: https://doi.org/10.1016/j.joms.2019.11.002
14. Sribnick EA, Sarda S, Sawvel MS, et al. Dog bite injuries in children: Clinical implications for head involvement. *Trauma* 2016;18(3):210-15. doi: 10.1177/1460408616631772
15. Kahn A, Bauche P, Lamoureux J, et al. Child victims of dog bites treated in emergency departments: a prospective survey. *European Journal of Pediatrics* 2003;162(4):254-58. doi: 10.1007/s00431-002-1130-6
16. Brogan TV, Bratton SL, Dowd MD, et al. SEVERE DOG BITES IN CHILDREN. *Pediatrics* 1995;96(5):947-50.
17. Westgarth C, Watkins F. Chapter 23: Impact of dog aggression on victims. In: Mills DS, Westgarth C, eds. Dog Bites: A Multidisciplinary Perspective. 1st Edition ed. Sheffield: 5M Publishing 2017:309-20.
18. Ji L, Xiaowei Z, Chuanlin W, et al. Investigation of posttraumatic stress disorder in children after animal-induced injury in China. *Pediatrics* 2010;126(2):e320-4. doi: 10.1542/peds.2009-3530 [published Online First: 2010/07/14]
19. Peters V, Sottiaux M, Appelboom J, et al. Posttraumatic stress disorder after dog bites in children. *The Journal of Pediatrics* 2004;144(1):121-22. doi: http://dx.doi.org/10.1016/j.jpeds.2003.10.024
20. Jakeman M, Oxley JA, Owczarczak-Garstecka SC, et al. Pet dog bites in children: management and prevention. *BMJ Paediatrics Open* 2020;4(1):e000726. doi: 10.1136/bmjpo-2020-000726
21. Hon KLE, Fu CCA, Chor CM, et al. Issues associated with dog bite injuries in children and adolescents assessed at the emergency department. *Pediatric Emergency Care* 2007;23(7):445-49.
22. Page MJ, McKenzie JE, Bossuyt PM, et al. The PRISMA 2020 statement: an updated guideline for reporting systematic reviews. *BMJ* 2021;372:n71. doi: 10.1136/bmj.n71
23. Albano AM, Miller PP, Zarate R, et al. Behavioral assessment and treatment of PTSD in prepubertal children: Attention to developmental factors and innovative strategies in the case study of a family. *Cognitive and Behavioral Practice* 1997;4(2):245-62. doi: https://doi.org/10.1016/S1077-7229(97)80003-X
24. Drell MJ, Gaensbauer TJ, Siegel CH, et al. Clinical round table: A case of trauma to a 21-month-old girl. *Infant Mental Health Journal* 1995;16(4):318-33. doi: https://doi.org/10.1002/1097-0355(199524)16:4<318::AID-IMHJ2280160407>3.0.CO;2-L
25. Gislason IL, Call JD. Dog Bite in Infancy: Trauma and Personality Development. *Journal of the American Academy of Child Psychiatry* 1982;21(2):203-07. doi: https://doi.org/10.1016/S0002-7138(09)60921-3
26. Macias D, Kwon DI, Walker PC, et al. Microvascular replantation of a composite facial avulsion in a 24-month-old child after dog bite. *Microsurgery* 2018;38(2):218-21. doi: https://doi.org/10.1002/micr.30275
27. Saha S. Life-Threatening Panfacial Wild Dog Bites in a Child. *Wilderness & Environmental Medicine* 2021;32(4):511-16. doi: https://doi.org/10.1016/j.wem.2021.07.001
28. Anyfantakis D, Botzakis E, Mplevrakis E, et al. Selective mutism due to a dog bite trauma in a 4-year-old girl: A case report. *Journal of Medical Case Reports* 2009;3

Confidential: For Review Only

29. de Carvalho MF, Hardtke LA, de Souza MF, et al. Comminuted mandibular fracture in child victim of dog bite. *Dent Traumatol* 2012;28(4):324-8. doi: 10.1111/j.1600-9657.2011.01079.x [published Online First: 2011/11/22]
30. Glasscock SE, MacLean Jr WE. Use of Contact Desensitization and Shaping in the Treatment of Dog Phobia and Generalized Fear of the Outdoors. *Journal of Clinical Child Psychology* 1990;19(2):169-71. doi: 10.1207/s15374424jccp1902_9
31. De Keuster T, Lamoureux J, Kahn A. Epidemiology of dog bites: A Belgian experience of canine behaviour and public health concerns. *Veterinary Journal* 2006;172(3):482-87.
32. Hersant B, Cassier S, Constantinescu G, et al. Facial dog bite injuries in children: Retrospective study of 77 cases. *Annales De Chirurgie Plastique Esthetique* 2012;57(3):230-39. doi: 10.1016/j.anplas.2011.11.003
33. Pédrono G, Ricard C, Bouilly M, et al. Assessment of 16-month Sequelae Due to Dog Bites Originally Studied in a French Multicenter Survey from 2009 to 2011. *Wounds* 2018;30(5):84-89. [published Online First: 2018/03/10]
34. Rossman BR, Bingham RD, Emde RN. Symptomatology and adaptive functioning for children exposed to normative stressors, dog attack, and parental violence. *J Am Acad Child Adolesc Psychiatry* 1997;36(8):1089-97. doi: 10.1097/00004583-199708000-00016 [published Online First: 1997/08/01]
35. Rusch MD, Grunert BK, Sanger JR, et al. Psychological adjustment in children after traumatic disfiguring injuries: A 12-month follow-up. *Plastic and Reconstructive Surgery* 2000;106(7):1451-58.
36. Touré G, Angoulangouli G, Méningaud J-P. Epidemiology and classification of dog bite injuries to the face: A prospective study of 108 patients. *Journal of Plastic, Reconstructive & Aesthetic Surgery* 2015;68(5):654-58. doi: https://doi.org/10.1016/j.bjps.2015.01.001
37. Boat BW, Dixon CA, Pearl E, et al. Pediatric dog bite victims: a need for a continuum of care. *Clin Pediatr (Phila)* 2012;51(5):473-7. doi: 10.1177/0009922811435504 [published Online First: 2012/02/02]
38. Schalamon J, Ainoedhofer H, Singer G, et al. Analysis of dog bites in children who are younger than 17 years. *Pediatrics* 2006;117(3):E374-E79. doi: 10.1542/peds.2005-1451
39. Shen J, Li S, Xiang H, et al. Antecedents and consequences of pediatric dog-bite injuries and their developmental trends: 101 cases in rural China. *Accident Analysis and Prevention* 2014;63:22-29.
40. Parent B, Bykowski MR, Marji FP, et al. Pediatric Craniofacial Fractures From Canine Bites. *J Craniofac Surg* 2021;32(4):1627-32. doi: 10.1097/scs.0000000000007546 [published Online First: 2021/03/21]
41. Doogan S, Thomas GV. Origins of fear of dogs in adults and children: The role of conditioning processes and prior familiarity with dogs. *Behaviour Research and Therapy* 1992;30(4):387-94. doi: https://doi.org/10.1016/0005-7967(92)90050-Q
42. Rusch MD, Gould LJ, Dzwierzynski WW, et al. Psychological Impact of Traumatic Injuries: What the Surgeon Can Do. *Plastic and Reconstructive Surgery* 2002;109(1):18-24.
43. Caffo E, Belaise C. Psychological aspects of traumatic injury in children and adolescents. *Child Adolesc Psychiatr Clin N Am* 2003;12(3):493-535. doi: 10.1016/s1056-4993(03)00004-x [published Online First: 2003/08/13]
44. Farrell LJ, Kershaw H, Ollendick T. Play-Modified One-Session Treatment for Young Children with a Specific Phobia of Dogs: A Multiple Baseline Case Series. *Child Psychiatry Hum Dev* 2018;49(2):317-29. doi: 10.1007/s10578-017-0752-x [published Online First: 2017/08/03]
45. Radtke SR, Muskett A, Coffman MF, et al. Bibliotherapy for Specific Phobias of Dogs in Young Children: A Pilot Study. *Journal of Child and Family Studies* 2023;32(2):373-83. doi: 10.1007/s10826-022-02304-2

Confidential: For Review Only

46. Oxley JA, Santa K, Meyer G, et al. A Systematic Scoping Review of Human-Dog Interactions in Virtual and Augmented Reality: The Use of Virtual Dog Models and Immersive Equipment. *Frontiers in Virtual Reality* 2022;3 doi: 10.3389/frvir.2022.782023
47. Farrell LJ, Miyamoto T, Donovan CL, et al. Virtual Reality One-Session Treatment of Child-Specific Phobia of Dogs: A Controlled, Multiple Baseline Case Series. *Behavior Therapy* 2021;52(2):478-91. doi: https://doi.org/10.1016/j.beth.2020.06.003

Confidential: For Review Only

FIGURES

Figure 1. Flow chart of study search and screening process for studies of psychological consequences of dog bites to children

Confidential: For Review Only

TABLES

Table 1: Case studies/small case-series identified in searches of psychological consequences of dog bites to children.

Paper title	Year	Author/s	Study design	Participants	Psychological findings
Behavioural Assessment and Treatment of PTSD in Prepubertal Children: Attention to Developmental Factors and Innovative Strategies in the Case Study of a Family	1997	AM Albano<br>PP Miller<br>G CotéDH Barlow	Mixed methods case study observing one family after a traumatic incident dog bite.<br>Prospective.<br>Diagnostic interviews.<br>Observational methods including rating sleep cycles of children, recording enuresis episodes and recording incidents of new behaviour since the incident.<br>Family behavioural tests with units of distress calculated.	N=4<br>M age 7<br>F age 6 (bitten)<br>F age 4<br>F age 2	Oldest 2 children diagnosed with PTSD.<br>Nocturnal changes in 2 children (bed wetting) represents a "significant change in functioning".<br>All 4 children experienced moderate to severe sleep disturbances.<br>"It was noted that as the age of the child increased, the more cognitively mediated symptoms and expression of fear became apparent."<br>Adult symptoms went into remission without treatment.
Selective mutism due to a dog bite trauma in a 4-year-old girl: a case report	2009	D Anyfantakis<br>E Botzakis<br>E Mplevrakis<br>EK Symvoulakis<br>I Arbiros	Case study.<br>Retrospective.	N=1<br>F age 5	On admission was confused and lethargic – findings showed hypovolemic shock.<br>2nd day of hospitalisation – child was withdrawn and depressed – used non verbal communication.<br>2 months after discharge was non-verbal and had reccurent traumatic memories.<br>Case met all criteria for selective mutism and acute PTSD.<br>Treatment consisted of "supportive psychotherapy for the child and consecutive sessions of counselling for her parents".<br>Improvement after 6M.
Clinical Round Table: A Case of Trauma to a 21-Month-Old Girl	1995	MJ Drell<br>TJ Gaensbauer<br>CH Siegel<br>M Sugar	Case study.<br>Retrospective.	N=1<br>F age 1	Detailed account of symptoms and therapy of child and mother.<br>Child displayed fearfulness of dogs, nightmares, anger, difficulty playing.

Confidential: For Review Only

Comminuted mandibular fracture in child victim of dog bite	2012	M Furtado de Carvalho<br>LAP Hardtke<br>MF Cota de Souza<br>V de Oliveira Araujo	Case study.<br>Retrospective.	N=1<br>M age 3	The patient was seen every day by oral and maxillofacial surgeons, dieticians, pediatricians, plastic surgeons, ophthalmologists, otorhinolaryngologists, physiotherapists, and psychologists."<br>Emphasis on the importance of a prompt multi-disciplinary approach.
CASE REPORT: Dog Bites in Infancy, Trauma and Personality Development	1982	IL Gislason<br>JD Call	Case series of children bitten by dogs.<br>Retrospective.<br>Interviewed accompanied by parents on 4 separate occasions.<br>Semi structured interview.	N=3<br>M age 1<br>F age 2<br>F age 3	"(first child) personality changed from being charming and outgoing prior to the bite to being cautious and inhibited after the bite."<br>First child referring to dog as "the bad dog" and mentioning wanting to shoot the dog when he grows up.<br>Clinginess increased in second child after incident.<br>Second child had nightmares for 3 years.<br>Third child developed bed wetting.<br>Third child showed increased clinginess, thumb sucking, nightmares.<br>Third child described as "withdrawn" after incident.<br>All children more afraid of strange dogs after their incidents.<br>All mothers were psychologically shaken.
Use of contact desensitization and shaping in the treatment of Dog Phobia and Generalized Fear of the Outdoors	2010	SE Glasscock<br>WE MacLean Jr	Case study.<br>Prospective.<br>Combination of contact desensitization, shaping and family counselling.	N=1<br>F age 6	"subsequently developed a phobic reaction to dogs and an avoidance of playing outdoors" after dog bite.<br>Consistent response to DS and shaping.<br>Study showed evidence that treatment needed maintenance to avoid reverting back.
Microvascular replantation of a composite facial avulsion in a 24-month-child after dog bite	2017	D Macias<br>DI Kwon<br>PC Walker<br>NR Peterson	Case study.<br>Retrospective.	N=1<br>F age 2	8 months post-op "The patient's mood and activity level had returned to pre-trauma baseline according to the patient's mother."
Life-Threatening Panfacial Wild Dog Bites in a Child	2021	S Saha	Case study.<br>Retrospective.	N=1<br>M age 1	"Psychologically, for the first 6 mo, he experienced severe bouts of panic attacks from loud noises,

Confidential: For Review Only

					especially the noises of lightning during storms." "He improved thereafter, and after 9 mo, while still avoiding all dogs, he would sometimes be fearful after hearing the noise of leaves rustling during high winds." "Otherwise, he recovered well under the supervision of a psychologist and a pediatrician."

Table 2 – Descriptive studies (larger than n=4), and cross-sectional studies, using both retrospective investigation and prospective follow-up, identified in searches of psychological consequences of dog bites to children.

Paper title	Year	Author/s	Study design	Participants	Psychological findings
Pediatric Dog Bite Victims: A Need for a Continuum of Care	2012	B Boat<br>C Dixon<br>E Pearl<br>L Thieken<br>SE Bucher	Descriptive study.<br>Prospective.<br>Inclusion criteria were 0Y-16Y with presenting complaint of "dog bite" to pediatric emergency department.<br>Parent completed a questionnaire after enrolment.<br>4 weeks later parents had 30 min phone interview.<br>Information obtained included: a) bite incident specifics, b) further medical/psychological care needed by child c) contact with additional agencies d) changes in the child's behaviour e) changes in parental concerns f) parent feedback on additional services	N=34<br>50 enrolled, 34 completed follow-up<br>Mean age 8yrs,<br>21 M, 13 F	No patients were hospitalised.<br>70% of victims demonstrated concerning behaviours since incident (most commonly talking a lot about the incident (29%), being fearful of dogs (27%), avoiding dogs (27%), being anxious or worried about seeing doctors or going to hospital (24%), being fearful (24%), having nightmares (21%).<br>Parents also reported concerns (eg. feeling guilty (59%), worried about scars (59), fearful for child's safety (44%).<br>No children received any psychological services.<br>50% of parents believed their children would benefit from interventions for help with fears.
Retrospective review of dog bite injuries in children presenting to a South Australian tertiary children's hospital emergency department	2014	SC Chiam<br>NS Solanki<br>M Lodge<br>M Higgins<br>AL Sparnon	Descriptive study.<br>Retrospective.<br>Review of children presenting to ED between 2009-2011.	N=277<br>Ages 0-17	2 children referred to a psychologist for management of post-traumatic stress.
Epidemiology of dog bites: A Belgian experience of	2006	T De Keuster<br>J Lamoureux<br>A Kahn	3 studies conducted in Belgium on bites in children, of which one relevant to our review.	Study 3<br>N=22	"Among the 22 children, 12 had shown symptoms of PTSD for more than one month; 5 had all

Confidential: For Review Only

canine behaviour and public health concerns			Descriptive study. Prospective. Questionnaire delivered to parents of child victims of dog bites, 2-9 months after minor surgical treatment.	Median age 7.5 (range 1-14 years). 12 M,10 F	DSM-IV criteria for PTSD and 7 had some but not all criteria". Symptoms included vivid recollection and re-living, avoidance behaviours or numbing, increased arousal, hypervigilance, nightmares, difficulties sleeping, aggression to siblings and peers. No difference was found for presence of PTSD symptoms in regard to age, gender, time since the bite, type of dog, place of the accident, owner of the dog, childs activity at the time of the accident, or part of the child's body bitten. "Only the children who suffered severe and/or multiple bites developed full PTSD symptoms".
Origins of fear of dogs in adults and children: The role of conditioning processes and prior familiarity with dogs	1991	S Doogan GV Thomas	Analytical cross-sectional. Retrospective. Questionnaire to "investigate fear of dogs". Adults and children asked to recall origin of their fear. Using Geer's FSS-2. Chi square testing for significance.	N=100 adults N=30 children (8-9 years)	"No difference in the frequency of being bitten or chased reported by fearful vs non fearful groups". "Significantly more fearful than non-fearful adults reported little contact with dogs prior to the onset of their fear (no significant difference in children)".
Facial dog bite injuries in children: Retrospective study of 77 cases	2012	B Hersant S Cassier G Constantinescu P Gavelle M -P Vazquez A Picard N Kadlub	Descriptive study. Retrospective. Study from 2002 to 2010. "We analyzed epidemiological, clinical data, surgical outcomes". Paper in French, only abstract available.	N=77 Mean age 5.36yrs.	35.1% of children had psychological problems afterward.
Investigation of Posttraumatic Stress Disorder in Children after Animal-Induced Injury in China	2010	L Ji Z Xiaowei W Chuanlin L Wei	Descriptive study. Prospective. Reviewed 358 charts and follow up data. "Family Apgar Scale assessment and PTSD screening were performed at ED admission". "On week 1 after ED admission the patient underwent evaluation for acute stress disorder diagnosis by using the Child Acute Stress Questionnaire". PTSD screening after 3M.	N=358 201 M 157 F Mean age 9.4 yrs	38 met symptom criteria for ASD(Acute Stress Disorder). 19 patients developed PTSD – 10 of which had major injuries. No significant differences regarding gender/age. ASD early predictor of PTSD.

Confidential: For Review Only

Child victims of dog bites treated in emergency departments: a prospective study	2003	A Kahn<br>P Bauche<br>J Lamoureux	Descriptive study.<br>Prospective.<br>Children with dog bites from 6 Eds.<br>Pediatricians and nurses collected standardized information via questionnaire.	N=100<br>Mean age 7.3 yrs<br>57 M, 43 F	Two children were treated by a psychologist: one child bitten on the face at home, the other bitten on the leg in a public place and left alone without care.
Pediatric Craniofacial Fractures from Canine Bites	2021	B Parent<br>MR Bykowski<br>FP Marji<br>S Ramgopal<br>JA Goldstein<br>JE Losee	Descriptive study.<br>Retrospective.<br>Included all pediatric dog bites between 2008-2019 at a single centre.<br>Selected cases with craniofacial fractures and described.	N=38 cases with craniofacial fractures described, of 3,602 paediatric dog bite encounters identified.<br>Age and gender for this group not stated.	Four patients (11%) subsequently diagnosed with PTSD, characterized mainly by recurring nightmares.<br>Two of these patients have required extensive counselling and anxiolytic medications.
Assessment of 16-month Sequelae Due to Dog Bites Originally Studied in a French Multicenter Survey from 2009 to 2011	2018	G Pedrono<br>C Ricard<br>M Bouilly<br>C Beata<br>G Sarcey<br>B Thelot	Descriptive study.<br>Prospective.<br>485 dog bites at 8 hospital EDs, followed up 16 M later (Sep 2010-Dec 2011).<br>Data collected by telephone or email	N=298<br>Mean age 28.1 yrs,<br>42% <15yrs	Adult and children findings not reported separately, however:<br>47% reported long term consequences, 16% of these being psychological<br>1/7 still experienced pain 16M after bite.<br>27% reported morale effected, higher for F (32%) than males (22%).<br><32% respondents reported being afraid of dogs after being bitten, slightly less frequent with children <15Y.
Posttraumatic Stress Disorder After Dog Bites in Children	2003	V Peters<br>M Sottiaux<br>J Appleboom<br>A Kahn	Descriptive study.<br>Prospective.<br>Between Apr 2001 – Feb 2002.<br>Child dog bite victims at pediatric ED .<br>Children under 16Y who received minor surgical treatment less than 48H after dog bite.<br>Parents agreed to do questionnaire 2-9M after bite.<br>Questionnaire with 60 items.<br>"Semistructured comprehensive interviews" by telephone.<br>8 questionnaires completed in further home visit.<br>"Statistical analyses were based on nonparametric	N=22<br>12 M, 10 F<br>Median age 7.5 yr	12/22 had PTSD symptoms for more than 1M, 5 had all DSM-IV criteria for PTSD; 7 children had some but not all criteria.<br>Symptoms included "vivid revivisecence of the traumatic event (12 children), avoidance behavior or numbing (7 children), and/or signs of increased arousal (6 children)".<br>"Only the children who had severe and/or multiple bites had full PTSD symptoms; none of the children with an accidental bite from their pet did so."

Confidential: For Review Only

			tests and x^2 tests with Yates correction for small series"		None received psychological support.
Symptomatology and Adaptive Functioning for Children Exposed to Normative Stressors, Dog Attack and Parental Violence	1997	BBR Rossman RD Bingham RN Emde	Descriptive study. Prospective. 86 children aged 4-9 "interviewed after 3 types of aversive experience: repetitive exposure to parental violence, a dog attack, or mild stressor". Parents completed (1) "family information and stressor structured interview", (2) "impact of event PTSD adult symptom questionnaire", (3) "PTSD Reaction Index", (4) "Child dissociative checklist", (5) "Life Events Questionnaire", (6) "Children's Behaviour Questionnaire", (7) "Child Behaviour Checklist". Children completed (1) "structured stressor interview", (2) "PTSD reaction index", (3) "Child Dissociative Index", (4) "Peabody Picture Vocabulary Test".	N=14 dog attack (DA) 10M 4F 4-6yrs n=4 7-9yrs n=10	"Parent report PTSD symptoms greater in both the DA and PV groups. Age and gender effects were significant for PTSD-RI(p), showing greater symptoms for younger children and girls. However, interactions of group with age and gender showed that the overall age and gender trends occurred mainly in the DA group. In contrast, a group by gender interaction for child-reported PTSD symptoms and reflected in the DA group showed boys reporting more symptoms than girls. Interpretation of this result is complicated by the fact that although boys reported greater PTSD symptoms, parents observed girls to show higher symptom levels. Mothers observed girls to have greater PTSD symptoms and boys to have greater dissociative symptoms, while child report showed boys to have greater PTSD symptoms. Mothers' PTSD symptoms significantly or marginally predicted greater child internalising behaviour problems and lower social competence."
Psychological Adjustment in Children after Traumatic Disfiguring Injuries: A 12-Month Follow-Up	2000	MD Rusch, BK Grunert JR Sanger WW Dzwierzynski H Matloub	Descriptive study. Prospective. Children treated in plastic and reconstructive surgery department for traumatic facial or extremity injuries, which included dog bites. Psychological evaluations performed within 4 days of injury. Interviews for children and parents (structured). Follow ups done at 1, 3, 6 and 12 month intervals.	N=57 Age 3-12 yrs 40 M, 17 F Not known which children suffered dog bites.	"Within 5 days of the traumatic event, 98 percent of the children were symptomatic for post traumatic stress disorder, depression or anxiety" (all but one child). "One month after the injury, 82 percent were symptomatic". "44 percent of the children continued to report symptoms at 12-month follow-up visits,

Confidential: For Review Only

			Categories assessed: reexperiencing symptoms, avoidance of thoughts/conversations promoting memory of incident, impaired attention/concentration, reaction to disfigurement, phantom pain.		and 21 percent met the diagnostic criteria for posttraumatic stress disorder". 37% children reported fear of dying. 75% children reported fear of re-injury. Children still experiencing fear of re-injury after 12M reduced partaking in "risky" or highly physical behaviours. No statistical significance in age. "82% of 17 girls (n=14) reported flashbacks at their 1-month follow up visits, compared with 45% of the 40 boys (n=18)(p=0.018). At the 12M follow up visits these differences no longer existed". "44% of our subjects exhibited significant long term psychological symptoms after their injuries that were directly attributable to their accidental injuries".
Analysis of Dog Bites in Children Who Are Younger Than 17 Years	2006	J Schalamon<br>H Ainoedhofer<br>G Singer<br>T Petnehazy<br>J Mayr<br>K Kiss<br>ME Hollwarth	Descriptive study. Retrospective/prospective. Review of medical charts of children under 17Y who sought medical attention for a dog bite between 1994-2003 at pediatric surgery department. Follow-up of 93% of the children 2-11 years after the dog attack (mean 7.2 years).	N=341<br>174 M, 167 F<br>Mean age 5.9yrs.	5 children complaining of nightmares after incidents. Fear of dogs remained in 34 children during long-term follow-up.
Antecedents and consequences of pediatric dog bite injuries and their developmental trends: 101 cases in rural China	2014	J Shen<br>S Li<br>H Xiang<br>S Lu<br>D C Schwebel	Descriptive study. Retrospective. All families had a dog bite victim under 18Y in the last 12 months. Structured interview with caregiver.	N=101<br>65% Male<br>Mean age 8.3 yrs	Children were reported to be generally unwilling to approach dogs after the incident and children tended to fear dogs to some degree after the incident.
Epidemiology and classification of dog bite injuries to the face: A prospective study of 108 patients	2015	G Toure<br>G Angoulangouli<br>J-P Meningaud	Descriptive study. Prospective. Study carried out over 13 years of dog bite intake to emergency surgeries. Information sheet filled out by victims bitten on the face.	108 incidents of dog bites (to face and neck) out of 13021 patients<br>49 M	23% encountered complications, of which 20% were psychological shock (anxiety and post-traumatic stress, sleep disorder, phobia of dogs and the patients own image).

Confidential: For Review Only

				~~59 F~~<br>~~69% <16yrs~~	

Confidential: For Review Only

Confidential: For Review Only

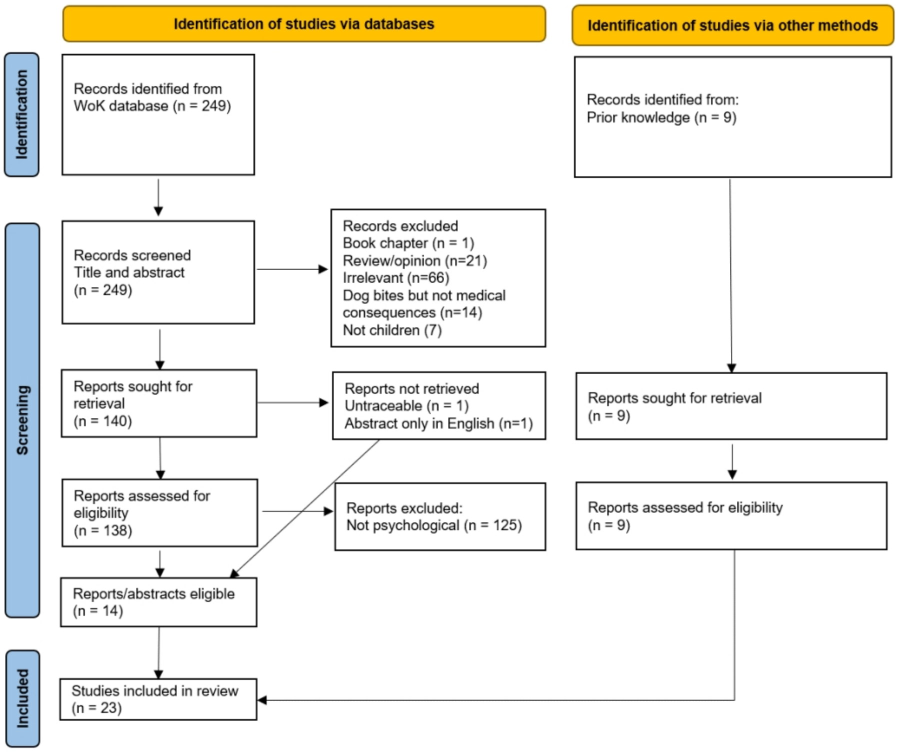

Figure 1. Flow chart of study search and screening process for studies of psychological consequences of dog bites to children

558x448mm (59 x 59 DPI)

Supplementary Material: A review of psychological effects of dog bites in children

Westgarth, Carri., Provozza, Serena., Nicholas, Jade., Gray, Victoria.

Confidential: For Review Only

Supplementary Table 1: Case studies/small case-series identified in searches of psychological consequences of dog bites to children.

**Paper title**	**Year**	**Author/s**	**Study design**	**Participants**	**Psychological findings**
Behavioural Assessment and Treatment of PTSD in Prepubertal Children: Attention to Developmental Factors and Innovative Strategies in the Case Study of a Family	1997	AM Albano<br>PP Miller<br>G Coté<br>DH Barlow	Mixed methods case study observing one family after a traumatic incident dog bite.<br>Prospective.<br>Diagnostic interviews.<br>Observational methods including rating sleep cycles of children, recording enuresis episodes and recording incidents of new behaviour since the incident.<br>Family behavioural tests with units of distress calculated.	N=4<br>M age 7<br>F age 6 (bitten)<br>F age 4<br>F age 2	Oldest 2 children diagnosed with PTSD.<br>Nocturnal changes in 2 children (bed wetting) represents a "significant change in functioning".<br>All 4 children experienced moderate to severe sleep disturbances.<br>"It was noted that as the age of the child increased, the more cognitively mediated symptoms and expression of fear became apparent."<br>Adult symptoms went into remission without treatment.
Selective mutism due to a dog bite trauma in a 4-year-old girl: a case report	2009	D Anyfantakis<br>E Botzakis<br>E Mplevrakis<br>EK Symvoulakis<br>I Arbiros	Case study.<br>Retrospective.	N=1<br>F age 5	On admission was confused and lethargic – findings showed hypovolemic shock.<br>2nd day of hospitalisation – child was withdrawn and depressed – used non verbal communication.<br>2 months after discharge was non-verbal and had reccurent traumatic memories.<br>Case met all criteria for selective mutism and acute PTSD.<br>Treatment consisted of "supportive psychotherapy for the child and consecutive sessions of counselling for her parents".<br>Improvement after 6M.

Clinical Round Table: A Case of Trauma to a 21-Month-Old Girl	1995	MJ Drell<br>TJ Gaensbauer<br>CH Siegel<br>M Sugar	Case study.<br>Retrospective.	N=1<br>F age 1	Detailed account of symptoms and therapy of child and mother.<br>Child displayed fearfulness of dogs, nightmares, anger, difficulty playing.
Comminuted mandibular fracture in child victim of dog bite	2012	M Furtado de Carvalho<br>LAP Hardtke<br>MF Cota de Souza<br>V de Oliveira Araujo	Case study.<br>Retrospective.	N=1<br>M age 3	The patient was seen every day by oral and maxillofacial surgeons, dieticians, pediatricians, plastic surgeons, ophthalmologists, otorhinolaryngologists, physiotherapists, and psychologists."<br>Emphasis on the importance of a prompt multi-disciplinary approach.
CASE REPORT: Dog Bites in Infancy, Trauma and Personality Development	1982	IL Gislason<br>JD Call	Case series of children bitten by dogs.<br>Retrospective.<br>Interviewed accompanied by parents on 4 separate occasions.<br>Semi structured interview.	N=3<br>M age 1<br>F age 2<br>F age 3	"(first child) personality changed from being charming and outgoing prior to the bite to being cautious and inhibited after the bite."<br>First child referring to dog as "the bad dog" and mentioning wanting to shoot the dog when he grows up.<br>Clinginess increased in second child after incident.<br>Second child had nightmares for 3 years.<br>Third child developed bed wetting.<br>Third child showed increased clinginess, thumb sucking, nightmares.<br>Third child described as "withdrawn" after incident.<br>All children more afraid of strange dogs after their incidents.<br>All mothers were psychologically shaken.
Use of contact desensitization and shaping in the treatment of Dog Phobia and Generalized Fear of the Outdoors	2010	SE Glasscock<br>WE MacLean Jr	Case study.<br>Prospective.<br>Combination of contact desensitization, shaping and family counselling.	N=1<br>F age 6	"subsequently developed a phobic reaction to dogs and an avoidance of playing outdoors" after dog bite.<br>Consistent response to DS and shaping.<br>Study showed evidence that treatment needed maintenance to avoid reverting back.
Microvascular replantation of a composite facial avulsion in a 24-month-child after dog bite	2017	D Macias<br>DI Kwon<br>PC Walker<br>NR Peterson	Case study.<br>Retrospective.	N=1<br>F age 2	8 months post-op "The patient's mood and activity level had returned to pre-trauma baseline according to the patient's mother."

Confidential: For Review Only

Life-Threatening Panfacial Wild Dog Bites in a Child	2021	S Saha	Case study. Retrospective.	N=1<br>M age 1	"Psychologically, for the first 6 mo, he experienced severe bouts of panic attacks from loud noises, especially the noises of lightning during storms."<br>"He improved thereafter, and after 9 mo, while still avoiding all dogs, he would sometimes be fearful after hearing the noise of leaves rustling during high winds."<br>"Otherwise, he recovered well under the supervision of a psychologist and a pediatrician."

Confidential: For Review Only

Supplementary Table 2 – Descriptive studies (larger than n=4), and cross-sectional studies, using only retrospective investigation identified in searches of psychological consequences of dog bites to children.

**Paper title**	**Year**	**Author/s**	**Study design**	**Participants**	**Psychological findings**
Retrospective review of dog bite injuries in children presenting to a South Australian tertiary children's hospital emergency department	2014	SC Chiam<br>NS Solanki<br>M Lodge<br>M Higgins<br>AL Sparnon	Descriptive study.<br>Retrospective.<br>Review of children presenting to ED between 2009-2011.	N=277<br>Ages 0-17	2 children referred to a psychologist for management of post-traumatic stress.
Origins of fear of dogs in adults and children: The role of conditioning processes and prior familiarity with dogs	1991	S Doogan<br>GV Thomas	Analytical cross-sectional.<br>Retrospective.<br>Questionnaire to "investigate fear of dogs".<br>Adults and children asked to recall origin of their fear.<br>Using Geer's FSS-2.<br>Chi square testing for significance.	N=100 adults<br>N=30 children (8-9 years)	"No difference in the frequency of being bitten or chased reported by fearful vs non fearful groups".<br>"Significantly more fearful than non-fearful adults reported little contact with dogs prior to the onset of their fear (no significant difference in children)".
Facial dog bite injuries in children: Retrospective study of 77 cases	2012	B Hersant<br>S Cassier G Constantinescu<br>P Gavelle<br>M -P Vazquez<br>A Picard<br>N Kadlub	Descriptive study.<br>Retrospective.<br>Study from 2002 to 2010.<br>"We analyzed epidemiological, clinical data, surgical outcomes".<br>Paper in French, only abstract available.	N=77<br>Mean age 5.36yrs.	35.1% of children had psychological problems afterward.
Pediatric Craniofacial Fractures from Canine Bites	2021	B Parent<br>MR Bykowski<br>FP Marji<br>S Ramgopal<br>JA Goldstein<br>JE Losee	Descriptive study.<br>Retrospective.<br>Included all pediatric dog bites between 2008-2019 at a single centre.<br>Selected cases with craniofacial fractures and described.	N=38 cases with craniofacial fractures described, of 3,602 paediatric dog bite encounters identified.	Four patients (11%) subsequently diagnosed with PTSD, characterized mainly by recurring nightmares.<br>Two of these patients have required extensive counselling and anxiolytic medications.

Confidential: For Review Only

				Age and gender for this group not stated.	
Antecedents and consequences of pediatric dog bite injuries and their developmental trends: 101 cases in rural China	2014	J Shen<br>S Li<br>H Xiang<br>S Lu<br>D C Schwebel	Descriptive study.<br>Retrospective.<br>All families had a dog bite victim under 18Y in the last 12 months.<br>Structured interview with caregiver.	N=101<br>65% Male<br>Mean age 8.3 yrs	Children were reported to be generally unwilling to approach dogs after the incident and children tended to fear dogs to some degree after the incident.

Confidential: For Review Only

Supplementary Table 3 – Descriptive studies (larger than n=4), and cross-sectional studies, using prospective follow-up, identified in searches of psychological consequences of dog bites to children.

Paper title	Year	Author/s	Study design	Participants	Psychological findings
Pediatric Dog Bite Victims: A Need for a Continuum of Care	2012	B Boat<br>C Dixon<br>E Pearl<br>L Thieken<br>SE Bucher	Descriptive study.<br>Prospective.<br>Inclusion criteria were 0Y-16Y with presenting complaint of "dog bite" to pediatric emergency department.<br>Parent completed a questionnaire after enrolment.<br>4 weeks later parents had 30 min phone interview.<br>Information obtained included: a) bite incident specifics, b) further medical/psychological care needed by child c) contact with additional agencies d) changes in the child's behaviour e) changes in parental concerns f) parent feedback on additional services	N=34<br>50 enrolled, 34 completed follow-up<br>Mean age 8yrs,<br>21 M, 13 F	No patients were hospitalised.<br>70% of victims demonstrated concerning behaviours since incident (most commonly talking a lot about the incident (29%), being fearful of dogs (27%), avoiding dogs (27%), being anxious or worried about seeing doctors or going to hospital (24%), being fearful (24%), having nightmares (21%).<br>Parents also reported concerns (eg. feeling guilty (59%), worried about scars (59), fearful for child's safety (44%).<br>No children received any psychological services.<br>50% of parents believed their children would benefit from interventions for help with fears.
Epidemiology of dog bites: A Belgian experience of canine behaviour and public health concerns	2006	T De Keuster<br>J Lamoureux<br>A Kahn	3 studies conducted in Belgium on bites in children, of which one relevant to our review.<br>Descriptive study.<br>Prospective.<br>Questionnaire delivered to parents of child victims of dog bites, 2-9 months after minor surgical treatment.	Study 3 N=22<br>Median age 7.5 (range 1-14 years).<br>12 M,10 F	"Among the 22 children, 12 had shown symptoms of PTSD for more than one month; 5 had all DSM-IV criteria for PTSD and 7 had some but not all criteria".<br>Symptoms included vivid recollection and re-living, avoidance behaviours or numbing, increased arousal, hypervigilance, nightmares, difficulties sleeping, aggression to siblings and peers.<br>No difference was found for presence of PTSD symptoms in regard to age, gender, time since the bite, type of dog, place of the accident, owner of the dog, childs activity at the time of the accident, or part of the child's body bitten.

Confidential: For Review Only

					"Only the children who suffered severe and/or multiple bites developed full PTSD symptoms".
Investigation of Posttraumatic Stress Disorder in Children after Animal-Induced Injury in China	2010	L Ji<br>Z Xiaowei<br>W Chuanlin<br>L Wei	Descriptive study.<br>Prospective.<br>Reviewed 358 charts and follow up data.<br>"Family Apgar Scale assessment and PTSD screening were performed at ED admission".<br>"On week 1 after ED admission the patient underwent evaluation for acute stress disorder diagnosis by using the Child Acute Stress Questionnaire".<br>PTSD screening after 3M.	N=358<br>201 M<br>157 F<br>Mean age 9.4 yrs	38 met symptom criteria for ASD(Acute Stress Disorder).<br>19 patients developed PTSD – 10 of which had major injuries.<br>No significant differences regarding gender/age.<br>ASD early predictor of PTSD.
Child victims of dog bites treated in emergency departments: a prospective study	2003	A Kahn<br>P Bauche<br>J Lamoureux	Descriptive study.<br>Prospective.<br>Children with dog bites from 6 Eds.<br>Pediatricians and nurses collected standardized information via questionnaire.	N=100<br>Mean age 7.3 yrs<br>57 M, 43 F	Two children were treated by a psychologist: one child bitten on the face at home, the other bitten on the leg in a public place and left alone without care.
Assessment of 16-month Sequelae Due to Dog Bites Originally Studied in a French Multicenter Survey from 2009 to 2011	2018	G Pedrono<br>C Ricard<br>M Bouilly<br>C Beata<br>G Sarcey<br>B Thelot	Descriptive study.<br>Prospective.<br>485 dog bites at 8 hospital EDs, followed up 16 M later (Sep 2010-Dec 2011).<br>Data collected by telephone or email	N=298<br>Mean age 28.1 yrs, 42% <15yrs	Adult and children findings not reported separately, however:<br>47% reported long term consequences, 16% of these being psychological<br>1/7 still experienced pain 16M after bite.<br>27% reported morale effected, higher for F (32%) than males (22%).<br><32% respondents reported being afraid of dogs after being bitten, slightly less frequent with children <15Y.
Posttraumatic Stress Disorder After Dog Bites in Children	2003	V Peters<br>M Sottiaux<br>J Appleboom<br>A Kahn	Descriptive study.<br>Prospective.<br>Between Apr 2001 – Feb 2002.<br>Child dog bite victims at pediatric ED .	N=22<br>12 M, 10 F<br>Median age 7.5 yr	12/22 had PTSD symptoms for more than 1M, 5 had all DSM-IV criteria for PTSD; 7 children had some but not all criteria.<br>Symptoms included "vivid reviviscence of the traumatic event (12 children), avoidance behavior or

Confidential: For Review Only

			Children under 16Y who received minor surgical treatment less than 48H after dog bite.<br>Parents agreed to do questionnaire 2-9M after bite.<br>Questionnaire with 60 items.<br>"Semistructured comprehensive interviews" by telephone.<br>8 questionnaires completed in further home visit.<br>"Statistical analyses were based on nonparametric tests and x^2 tests with Yates correction for small series"		numbing (7 children), and/or signs of increased arousal (6 children)".<br>"Only the children who had severe and/or multiple bites had full PTSD symptoms; none of the children with an accidental bite from their pet did so."<br>None received psychological support.
Symptomatology and Adaptive Functioning for Children Exposed to Normative Stressors, Dog Attack and Parental Violence	1997	BBR Rossman<br>RD Bingham<br>RN Emde	Descriptive study.<br>Prospective.<br>86 children aged 4-9 "interviewed after 3 types of aversive experience: repetitive exposure to parental violence, a dog attack, or mild stressor".<br>Parents completed (1) "family information and stressor structured interview", (2) "impact of event PTSD adult symptom questionnaire", (3) "PTSD Reaction Index", (4) "Child dissociative checklist", (5) "Life Events Questionnaire", (6) "Children's Behaviour Questionnaire", (7) "Child Behaviour Checklist".<br>Children completed (1) "structured stressor interview", (2) "PTSD reaction index", (3) "Child Dissociative Index", (4) "Peabody Picture Vocabulary Test".	N=14 dog attack (DA)<br>10M 4F<br>4-6yrs n=4<br>7-9yrs n=10	"Parent report PTSD symptoms greater in both the DA and PV groups.<br>Age and gender effects were significant for PTSD-Rl(p), showing greater symptoms for younger children and girls. However, interactions of group with age and gender showed that the overall age and gender trends occurred mainly in the DA group. In contrast, a group by gender interaction for child-reported PTSD symptoms and reflected in the DA group showed boys reporting more symptoms than girls. Interpretation of this result is complicated by the fact that although boys reported greater PTSD symptoms, parents observed girls to show higher symptom levels.<br>Mothers observed girls to have greater PTSD symptoms and boys to have greater dissociative symptoms, while child report showed boys to have greater PTSD symptoms.<br>Mothers' PTSD symptoms significantly or marginally predicted greater child internalising behaviour problems and lower social competence."

Confidential: For Review Only

Psychological Adjustment in Children after Traumatic Disfiguring Injuries: A 12-Month Follow-Up	2000	MD Rusch,<br>BK Grunert<br>JR Sanger<br>WW Dzwierzynski<br>H Matloub	Descriptive study.<br>Prospective.<br>Children treated in plastic and reconstructive surgery department for traumatic facial or extremity injuries, which included dog bites.<br>Psychological evaluations performed within 4 days of injury.<br>Interviews for children and parents (structured).<br>Follow ups done at 1, 3, 6 and 12 month intervals.<br>Categories assessed: reexperiencing symptoms, avoidance of thoughts/conversations promoting memory of incident, impaired attention/concentration, reaction to disfigurement, phantom pain.	N=57<br>Age 3-12 yrs<br>40 M, 17 F<br>Not known which children suffered dog bites.	"Within 5 days of the traumatic event, 98 percent of the children were symptomatic for post traumatic stress disorder, depression or anxiety" (all but one child).<br>"One month after the injury, 82 percent were symptomatic".<br>"44 percent of the children continued to report symptoms at 12-month follow-up-visits, and 21 percent met the diagnostic criteria for posttraumatic stress disorder".<br>37% children reported fear of dying.<br>75% children reported fear of re-injury.<br>Children still experiencing fear of re-injury after 12M reduced partaking in "risky" or highly physical behaviours.<br>No statistical significance in age.<br>"82% of 17 girls (n=14) reported flashbacks at their 1-month follow up visits, compared with 45% of the 40 boys (n=18)(p=0.018). At the 12M follow up visits these differences no longer existed".<br>"44% of our subjects exhibited significant long term psychological symptoms after their injuries that were directly attributable to their accidental injuries".
Analysis of Dog Bites in Children Who Are Younger Than 17 Years	2006	J Schalamon<br>H Ainoedhofer<br>G Singer<br>T Petnehazy<br>J Mayr<br>K Kiss<br>ME Hollwarth	Descriptive study.<br>Retrospective/prospective.<br>Review of medical charts of children under 17Y who sought medical attention for a dog bite between 1994-2003 at pediatric surgery department.<br>Follow-up of 93% of the children 2-11 years after the dog attack (mean 7.2 years).	N=341<br>174 M, 167 F<br>Mean age 5.9yrs.	5 children complaining of nightmares after incidents.<br>Fear of dogs remained in 34 children during long-term follow-up.
Epidemiology and classification of dog bite injuries to the face: A prospective study of 108 patients	2015	G Toure<br>G Angoulangouli<br>J-P Meningaud	Descriptive study.<br>Prospective.<br>Study carried out over 13 years of dog bite intake to emergency surgeries.<br>Information sheet filled out by victims bitten on the face.	108 incidents of dog bites (to face and neck) out of 13021 patients<br>49 M<br>59 F	23% encountered complications, of which 20% were psychological shock (anxiety and post-traumatic stress, sleep disorder, phobia of dogs and the patients own image).

Confidential: For Review Only

				69% <16yrs	

Confidential: For Review Only
